# Bioorthogonal chemistry for deciphering lipid biology in living systems

**DOI:** 10.1039/d6cb00259e

**Published:** 2026-09-23

**Authors:** Brenda Portasany, Federica A. Souto-Trinei, Roberto J. Brea

**Affiliations:** a Bioinspired Nanochemistry (BioNanoChem) Group, CICA - Centro Interdisciplinar de Química e Bioloxía, Departamento de Química, Facultad de Ciencias, Universidade da Coruña, Rúa As Carballeiras s/n, Campus de Elviña 15071 A Coruña Spain roberto.brea@udc.es; b Grupo de Nanotecnología Traslacional (NANOTx), Instituto de Investigación Biomédica de A Coruña (INIBIC) Avenida Xubias de Arriba 84 15006 A Coruña Spain

## Abstract

Lipids are among the most structurally diverse classes of biomolecules and play fundamental roles in membrane organization, energy storage, and cellular signalling. However, unlike proteins and nucleic acids, they are not directly encoded by the genome, making their selective visualization, manipulation, and functional interrogation in living systems particularly challenging. Over the past two decades, bioorthogonal chemistry has transformed lipid research by enabling the incorporation of minimally perturbing chemical reporters and the use of highly selective ligation reactions in complex biological environments. These advances have made it possible to synthesize, label, image, quantify, and manipulate lipids with unprecedented spatial and temporal precision. In this review, we discuss how bioorthogonal chemistry has expanded the experimental toolkit for deciphering lipid biology, highlighting applications ranging from the *in situ* synthesis of membrane-forming lipids and live-cell imaging to the investigation of lipid–protein interactions and the development of bioinspired therapeutic strategies. Finally, we discuss the remaining challenges and emerging opportunities for achieving a quantitative, dynamic, and spatially resolved understanding of lipid function in living systems.

## Abstract 2

Bioorthogonal chemistry has transformed lipid research by enabling precise synthesis, labelling, imaging, and manipulation of lipids in living cells, advancing the study of lipid function, dynamics, signalling, and interactions.

## Graphical abstract

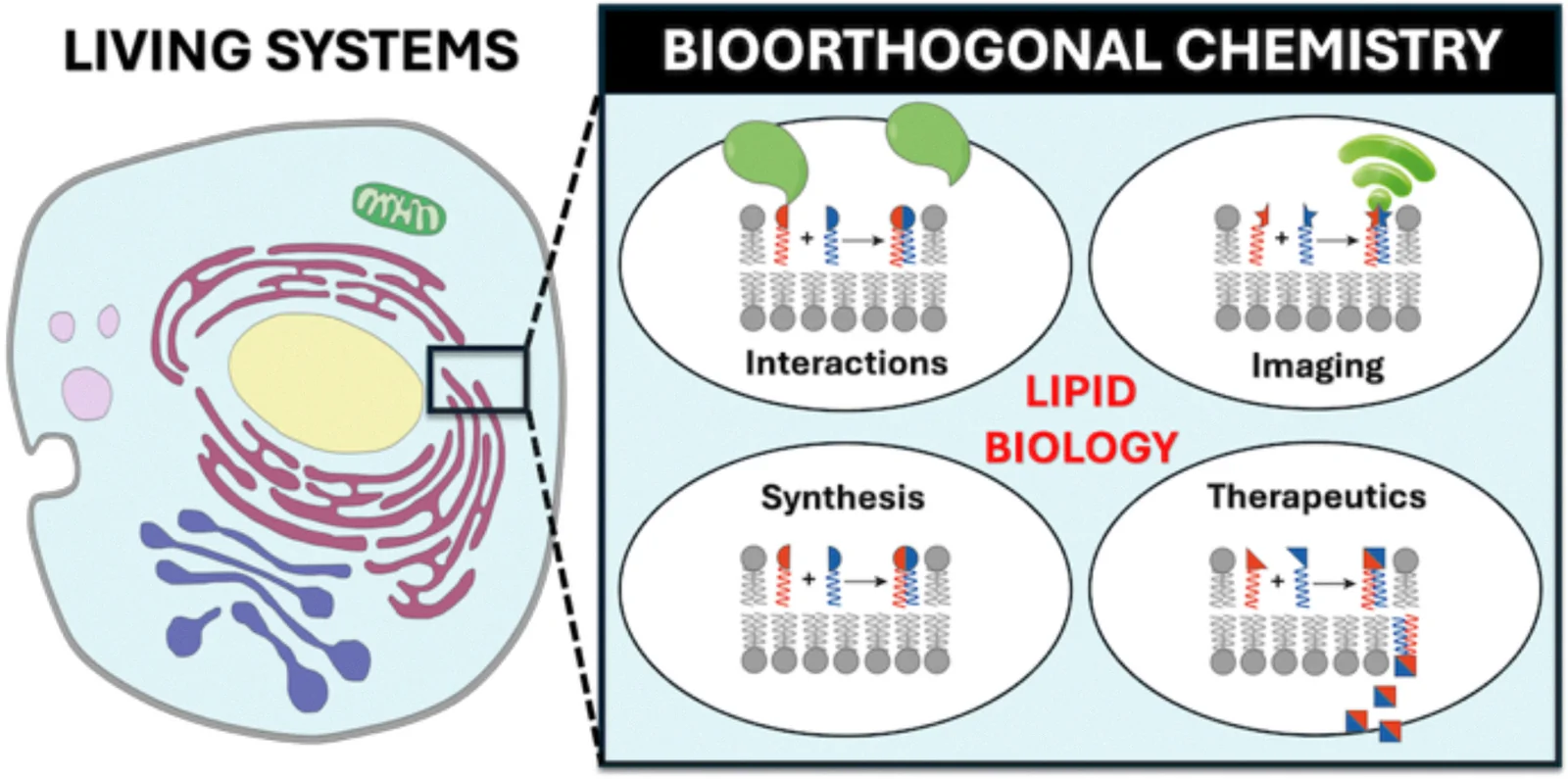

## 1. Introduction

Lipids constitute the structural framework of all biological membranes and represent one of the most chemically diverse and functionally versatile classes of biomolecules in living systems.^1,2^ Beyond defining the boundaries of cells and organelles, they serve as energy-storage molecules, signalling mediators, and key regulators of a wide range of physiological processes, including cell proliferation, apoptosis, immune responses, and neuronal communication.^1–3^ Despite their central biological importance, our understanding of how lipid diversity is generated, spatially organised within membranes, and recognised by lipid-binding proteins has historically lagged behind that of proteins and nucleic acids. This disparity arises from a fundamental challenge: unlike proteins and nucleic acids, lipids are not directly encoded by genes and therefore cannot be readily manipulated using conventional genetic approaches. Moreover, their relatively small size makes them particularly susceptible to experimental perturbation, since fluorophore conjugation can significantly alter their structure, physicochemical properties, intracellular trafficking, and biological function.^1,2,4^

Bioorthogonal chemistry has transformed lipid research by enabling highly selective chemical reactions that proceed efficiently in complex biological environments without interfering with native cellular processes.^5^ The central concept relies on the incorporation of a minimally perturbing chemical reporter – such as an azide, terminal alkyne, strained alkene, tetrazine, or Raman-active functional group – into a lipid or its metabolic precursor. These compact tags are designed to preserve the physicochemical properties of the parent molecule while providing a selective handle for subsequent reaction with complementary probes.^2,5^ Originally developed for the study of glycans and proteins,^6^ this two-step strategy decouples lipid delivery from downstream detection, isolation, or functional manipulation, offering exceptional flexibility for investigating lipid metabolism, trafficking, localization, and molecular interactions in living systems.^6^

Bioorthogonal reporters can be introduced either through the chemical synthesis of lipid analogues or by metabolic labelling, in which tagged precursors are processed by endogenous biosynthetic pathways and incorporated into native lipid species.^1,5^ These complementary approaches enable the visualization and functional interrogation of lipids with high spatial and temporal resolution while minimizing perturbation of endogenous lipid metabolism. Over the past two decades, the scope of bioorthogonal chemistry has expanded far beyond its original applications in imaging. It is now routinely employed to synthesize membrane-forming lipids *in situ*, capture transient lipid–protein interactions, investigate membrane organization and dynamics, and engineer lipid-based therapeutic and delivery platforms.^2,3,7–11^ Collectively, these advances have established bioorthogonal chemistry as a cornerstone of modern lipid chemical biology.

In this review, we examine how bioorthogonal chemistry has enabled the study, manipulation, and analysis of lipids across multiple levels of biological organization. We first discuss bioorthogonal strategies for the *in situ* synthesis of lipids and the generation of membrane-bound systems. We then review approaches for lipid labelling and imaging, including both synthetic probes and metabolic labelling methodologies. Next, we highlight applications in the investigation of lipid–protein interactions and membrane-associated biological processes. Finally, we discuss emerging therapeutic and translational applications, emphasizing how bioorthogonal chemistry is increasingly being used not only to observe lipid biology but also to actively control and engineer it.

## 2. Synthetic approaches to membrane-forming lipids

The abiotic synthesis of membrane-forming lipids under aqueous and physiologically relevant conditions remains a central challenge in biomimetic membrane chemistry and bottom-up synthetic biology. In living organisms, phospholipid biosynthesis is orchestrated by tightly regulated membrane-associated enzymatic pathways that coordinate acyl transfer, headgroup modification, and membrane remodelling,^12,13^ making these processes difficult to reproduce *in vitro*. Consequently, considerable efforts have focused on developing chemoselective and bioorthogonal reactions capable of generating membrane-forming amphiphiles *in situ* from simple, non-membranous precursors.^2,7,14–19^ Beyond offering alternative routes to lipid synthesis, these strategies provide powerful tools to investigate membrane biogenesis, manipulate membrane composition, and control lipid-dependent processes with spatiotemporal precision.^7,8,15–17^ A particularly attractive feature is that the reaction products frequently undergo spontaneous self-assembly into membrane-like architectures, directly linking selective covalent bond formation with the emergence of higher-order supramolecular structures.^7,15–17^

### 2.1 KAHA and KAT ligation

Among the available strategies, hydroxylamine-based ligations have emerged as particularly powerful methodologies for the nonenzymatic synthesis of phospholipid analogues in aqueous media. In particular, α-ketoacid-hydroxylamine (KAHA)^20^ and potassium acyltrifluoroborate (KAT)^21^ ligations enable efficient amide bond formation under mild aqueous conditions without the need for additional activating agents or catalysts. These reactions rely on the bioorthogonal coupling of hydroxylamine-functionalised substrates with activated acyl donors: α-ketoacids in KAHA ligation and acyltrifluoroborates in KAT ligation. Initially developed for peptide synthesis and bioconjugation,^20,22,23^ both reactions have since been adapted for lipid chemistry owing to their excellent compatibility with aqueous media, broad functional-group tolerance, and high chemoselectivity under biologically relevant conditions.^7^

The potential of these ligation chemistries for membrane construction was elegantly demonstrated by Chen *et al.*, who reported the rapid formation of noncanonical phospholipids through the reaction of hydroxylamine-functionalised lysophospholipids (HA–LPs) with either α-ketoacids (KAs) or potassium acyltrifluoroborates (KATs) (Fig. 1A).^7^ In this system, the ligation directly generates amidophospholipids (APLs) under physiological conditions, bypassing the enzymatic acyl-transfer reactions that normally govern phospholipid biosynthesis. The resulting amphiphiles retained the characteristic self-assembly behaviour of natural phospholipids and spontaneously formed giant vesicles resembling biological membranes. These findings showed that simple, water-soluble precursors can be transformed into membrane-forming amphiphiles through a single bioorthogonal ligation, providing a straightforward route to *de novo* membrane assembly under physiological conditions. Moreover, both KAHA and KAT ligations proceeded efficiently in aqueous media while maintaining sufficient chemoselectivity to operate in complex biological environments. Notably, both strategies proved compatible with living cells. Following incubation of HeLa cells with the corresponding precursors, *in situ* formation of amidophospholipids was confirmed by mass spectrometry (Fig. 1B), while confocal microscopy revealed membrane protrusions and vesicle-like budding events associated with localised lipid production (Fig. 1C). These observations indicate that hydroxylamine-based ligations can not only generate membrane-forming lipids within cells but also trigger membrane-remodelling processes, highlighting their potential as abiotic tools for engineering cellular membranes.

**Fig. 1:**
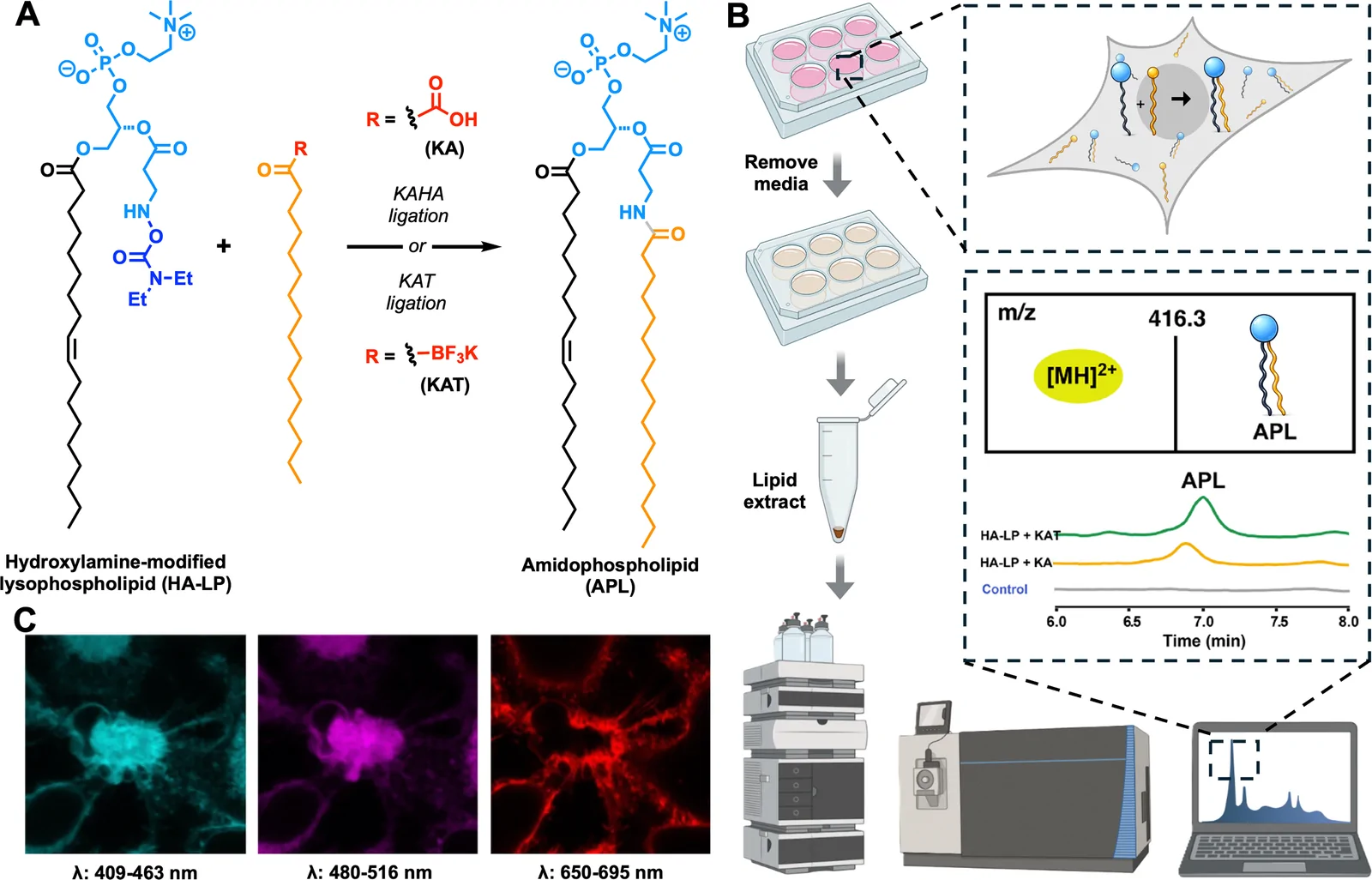
KAHA and KAT ligations in living cells.^7^ (A) *De novo* formation of membrane-forming phospholipids *via* KAHA and KAT ligations. (B) Schematic overview of the *in vivo* experiments performed in HeLa cells. (C) Live-cell confocal microscopy images revealing vesicular membrane structures budding from the plasma membrane. Images were acquired at 409–463 nm (cyan) for the lipid-ordered channel, 480–516 nm (magenta) for the lipid-disordered channel, and 650–695 nm (red) for the plasma membrane stain. Figure adapted from ref. 7 with permission from John Wiley & Sons, copyright 2024.

### 2.2 Photocatalytic decarboxylative alkylation

Although hydroxylamine-based ligations enable the efficient generation of membrane-forming amphiphiles under biologically relevant conditions, they rely on constitutively reactive precursors and predominantly yield noncanonical amidophospholipids. These limitations have motivated the development of alternative strategies capable of producing structurally native phospholipids while providing external control over the timing and localization of lipid formation. In this context, visible-light-mediated photocatalytic decarboxylative alkylation has emerged as a powerful platform for constructing C–C bonds under mild, biocompatible conditions.^24^

The potential of this strategy for membrane engineering was demonstrated by Devaraj and co-workers, who reported the visible-light-driven synthesis of natural phospholipids in both artificial membrane systems and living cells (Fig. 2).^15^ In their photoredox lipid ligation (PLL) strategy, alkene-functionalised lysophospholipids were coupled with fatty acid-derived *N*-hydroxyphthalimide (NHPI) esters under photoredox conditions, generating phospholipids through direct C–C bond formation (Fig. 2A). In contrast to hydroxylamine-based ligations, which afford amidophospholipid analogues, this method produces phospholipids with native architectures without introducing artificial linkages into the membrane scaffold. Time-lapse phase-contrast microscopy further demonstrated the *de novo* formation of phospholipid vesicles under localized green-light irradiation, revealing vesicle growth, budding, and division as the reaction progressed (Fig. 2B).

**Fig. 2:**
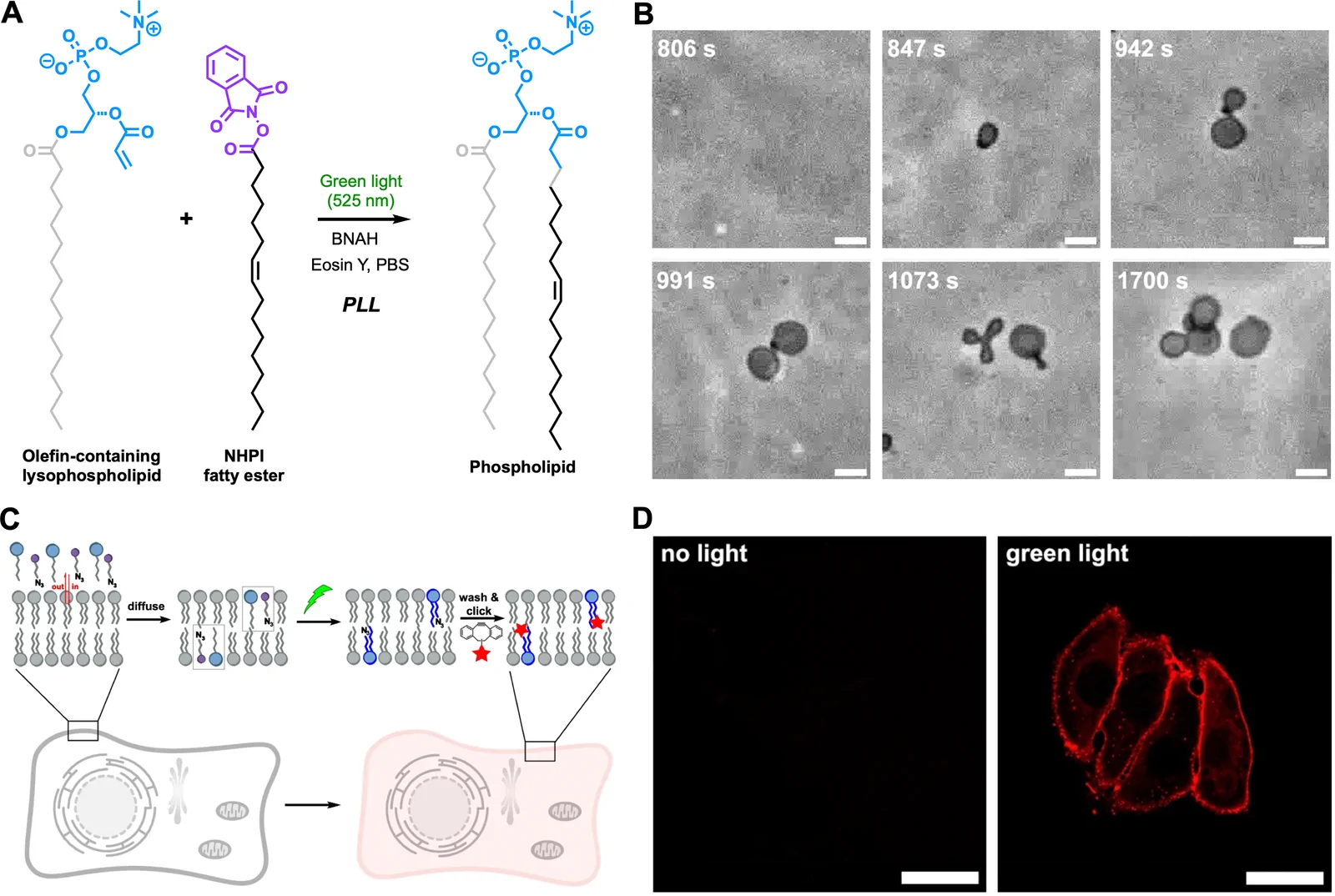
Photoredox lipid ligation (PLL).^15^ (A) *In situ* generation of phospholipids *via* a photocatalytically driven decarboxylative alkylation between an alkene-functionalized lysophospholipid and an *N*-hydroxyphthalimide (NHPI) fatty ester. (B) Time-lapse phase-contrast microscopy images illustrating the *de novo* formation of phospholipid vesicles under localized green light irradiation (530–580 nm). Vesicle growth, budding, and division are observed throughout the irradiation period. Scale bars: 5 µm. (C) Schematic overview of PLL in living cells. (D) Representative confocal microscopy images of HeLa cells treated with olefin-containing lysophospholipid, azido-NHPI fatty ester, BNAH (1-benzyl-1,4-dihydronicotinamide), and eosin Y, either maintained in the dark (left) or exposed to 525 nm green light (right) for 5 min. Following irradiation and washing, cells were incubated with dibenzocyclooctyne (DBCO)-Fluor 594, washed again, and visualized by confocal microscopy. Scale bars: 50 µm. Figure adapted from ref. 15 under a Creative Commons Attribution 4.0 International License.

The methodology was also shown to operate in living cells (Fig. 2C and D). Following incubation of mammalian cells with the corresponding precursors, visible-light irradiation led to the formation of phospholipids, as confirmed by mass spectrometry and lipidomic analyses. The newly formed phospholipids were additionally visualised by post-irradiation labelling of membrane-incorporated azide groups with the fluorescent probe dibenzocyclooctyne (DBCO)-Fluor 594, confirming light-dependent phospholipid formation at the plasma membrane (Fig. 2C and D). Confocal microscopy further revealed localised membrane-remodelling, including membrane protrusions and vesicle-like budding events, indicating that photocatalytic phospholipid synthesis can promote membrane growth directly within cellular environments while maintaining excellent biocompatibility.

Photocatalytic decarboxylative alkylation substantially expands the bioorthogonal toolbox for membrane engineering, illustrating how abiotic reaction networks can be harnessed not only to construct membranes but also to control their growth, remodelling, and function with unprecedented precision. At the same time, this approach underscores the next frontier in the field: moving beyond phospholipid synthesis toward the *in situ* generation of specific lipid subclasses with defined biological roles.

### 2.3 Traceless ceramide ligation

Ceramides (Cers) constitute a structurally distinct class of membrane lipids that play essential roles in membrane organization, lipid-domain formation, and cellular signalling.^25–28^ Beyond serving as central intermediates in sphingolipid metabolism, they regulate membrane curvature, permeability, and numerous cellular processes, including apoptosis, differentiation, and stress responses.^8,25–28^ Despite their biological importance, the selective synthesis of defined ceramide species in biological environments remains challenging because of the complexity of sphingolipid metabolic networks and the limited compatibility of conventional synthetic methodologies with aqueous and cellular systems.^25–28^

To overcome these limitations, Rudd *et al.* developed traceless ceramide ligation (TCL), a chemoselective strategy that enables the direct formation of native ceramides from membrane-associated precursors under physiological conditions (Fig. 3).^8^ In this approach, fatty acid salicylaldehyde (FAS) esters react selectively with sphingosine (Sph) through condensation with its characteristic 1,2-amino alcohol motif, followed by an intramolecular acyl transfer that generates the corresponding ceramide while releasing the salicylaldehyde auxiliary. Because no residual functional groups remain after ligation, the products are chemically indistinguishable from naturally occurring ceramides. This traceless outcome represents a key advantage over many bioorthogonal ligation strategies, which typically leave behind non-native linkages or persistent chemical handles in the final lipid structure.^3^

**Fig. 3:**
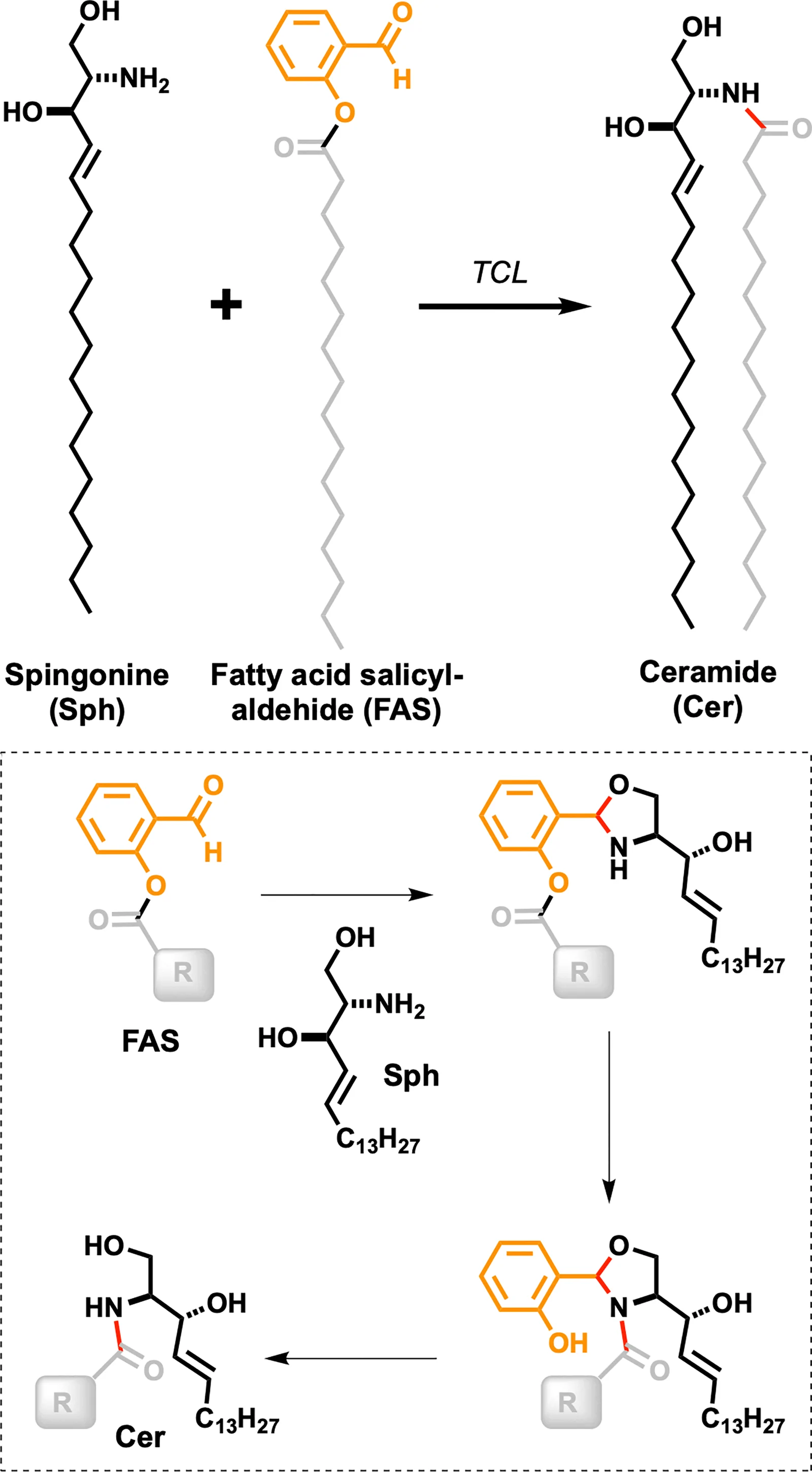
Traceless ceramide ligation (TCL).^8^ Top, Synthesis of ceramides (Cers) *via* TCL between sphingosine (Sph) and fatty acid salicylaldehyde (FAS) esters. Bottom, Mechanism of the reaction.

TCL proved effective in both model membrane systems and living cells. Following incubation of mammalian cells with the corresponding precursors, the formation of defined ceramide species was confirmed by mass spectrometry. By generating ceramides directly within cellular membranes, TCL circumvents many of the limitations associated with exogenous lipid delivery or genetic manipulation of sphingolipid biosynthesis, providing a unique platform for probing structure–function relationships while preserving the native membrane environment.

Importantly, TCL enables not only the synthesis of ceramides but also the direct interrogation of their biological functions. Using this approach, Devaraj and co-workers demonstrated that saturated and unsaturated ceramide species display distinct apoptotic activities, revealing how subtle structural variations translate into markedly different cellular responses.^29^ More broadly, these studies established that chemoselective lipid synthesis can be used to manipulate membrane composition with molecular precision, providing a powerful approach for dissecting the relationship between membrane lipid composition and cellular function.

### 2.4 Other methodologies

While the strategies described in the previous sections represent key advances in *in situ* lipid synthesis, a variety of complementary chemical methodologies have also contributed to the development of membrane-generating reaction systems.^8,26–28^ Although many of these approaches have not yet reached the same level of structural precision or biological compatibility as the aforementioned strategies, they collectively highlight the remarkable diversity of chemical transformations capable of producing membrane-forming amphiphiles under abiotic conditions. As a result, the construction of functional membrane systems is no longer limited to a small number of chemoselective ligation reactions but instead encompasses an expanding repertoire of bioorthogonal, photochemical, and dynamic covalent processes. Together, these developments underscore the growing ability of synthetic chemistry to regulate membrane formation, composition, and function through programmable reaction networks.

Among these methodologies, click chemistry has played a particularly prominent role in the synthesis of phospholipid analogues and membrane-active amphiphiles. Since the introduction of copper-catalysed azide–alkyne cycloaddition (CuAAC), its exceptional chemoselectivity, efficiency, and modularity have been widely exploited to generate triazole-containing phospholipids and glycolipids.^16,17,30^ In many cases, these synthetic amphiphiles retain their ability to self-assemble into bilayer structures, demonstrating that non-native chemical linkages can preserve the fundamental physicochemical properties required for membrane formation.^16,17^ Furthermore, the development of strain-promoted azide–alkyne cycloaddition (SPAAC) and other copper-free click reactions has extended the applicability of these approaches to biological systems, enabling membrane labelling, lipid tracking, and *in situ* membrane modification under mild and biocompatible conditions.^31^ These studies established click chemistry as one of the earliest and most versatile platforms for membrane engineering and provided a foundation for the broader application of bioorthogonal reactions to manipulate lipid organization and membrane function in complex biological environments. Nevertheless, several of these click-based strategies still leave a triazole or other non-native moiety in the final lipid product, underscoring the continued need for bioorthogonal chemistries that preserve native lipid connectivity and functionality while maintaining high chemoselectivity.

## 3. Imaging and visualization of cellular lipids

Lipids present unique challenges for molecular visualization that distinguish them from proteins and nucleic acids. Unlike proteins, which can be genetically tagged through fluorescent fusion strategies, or nucleic acids, which can be modified through sequence-specific approaches, lipids are not directly encoded by genes and therefore cannot be readily manipulated using conventional biological tools. Moreover, indirect approaches targeting lipid biosynthetic enzymes often affect multiple lipid species simultaneously, limiting molecular resolution.^1,2^

Additional challenges arise from the physicochemical properties of lipids themselves. Due to their small size and amphiphilic nature, direct conjugation of bulky fluorophores can substantially alter lipid polarity, membrane partitioning, trafficking, and biological activity.^5,21^ Bioorthogonal chemistry has provided a powerful solution to this problem by enabling the incorporation of minimally perturbing chemical reporters that can be selectively converted into fluorescent signals through rapid and selective reactions in biological environments.^1–3,5,32–38^ Two complementary strategies have emerged: synthetic lipid probes, in which a bioorthogonal handle is incorporated directly into a lipid analogue,^32,39,40^ and metabolic labelling approaches, in which modified precursors are processed by endogenous pathways and incorporated into native lipid pools.^1,9,41–49^ Together, these approaches have transformed the visualization and manipulation of cellular lipids with unprecedented spatial and temporal precision.

### 3.1 Synthetic lipid probes

The development of synthetic lipid probes has been guided by the principle that chemical reporters should minimally perturb the structure and function of the parent lipid. This is commonly achieved through a two-step strategy in which a small bioorthogonal handle is introduced into a lipid analogue and subsequently reacted with a fluorescent reporter after cellular incorporation. By separating lipid recognition from fluorescence generation, this approach avoids the substantial perturbations associated with directly fluorophore-labelled lipids and enables more faithful visualization of lipid localization and dynamics.^2,5^

The first demonstration of bioorthogonal lipid imaging in living systems employed a phosphatidic acid (PA) analogue containing a terminal alkyne reporter that could be selectively conjugated to an azide-functionalised fluorophore through CuAAC (Fig. 4A).^32^ The minimal structural perturbation introduced by the alkyne allowed the modified lipid to closely mimic endogenous PA behaviour. Importantly, the intracellular distribution of the bioorthogonally labelled lipid differed from that of directly fluorescent analogues, establishing a fundamental principle for lipid imaging: small chemical reporters preserve biological function more effectively than directly attached fluorophores.^32^

**Fig. 4:**
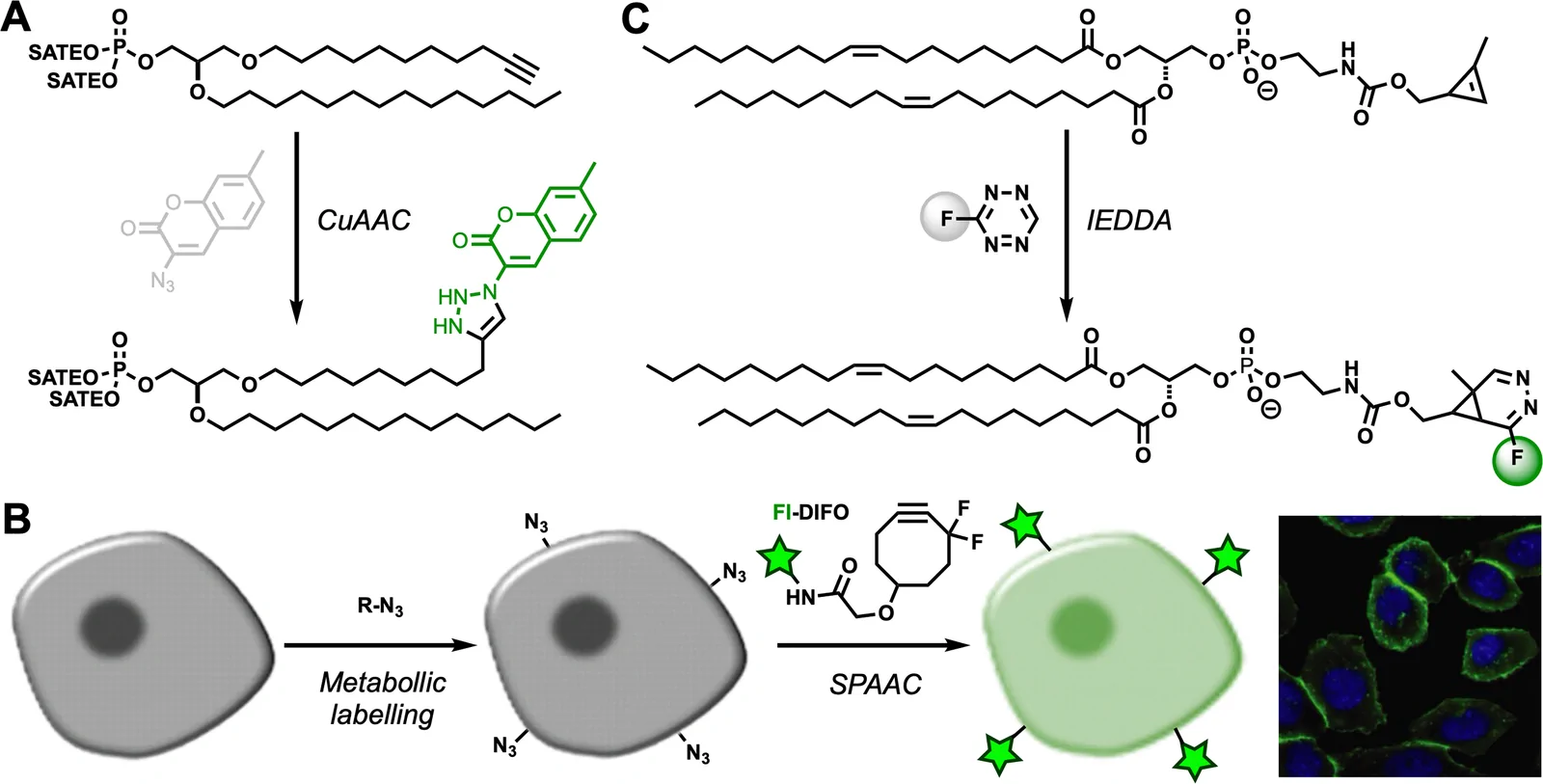
Bioorthogonal lipid labelling strategies using synthetic lipid probes. (A) Alkyne-containing PA undergoes CuAAC with an azide-functionalised fluorophore to generate fluorescently labelled lipids [SATE: S-acyl-2-thioethyl].^32^ (B) Metabolic labelling and subsequent detection of cell-surface biomolecules by SPAAC using difluorinated cyclooctyne (DIFO)-based fluorophores. Figure adapted from ref. 33 with permission from the National Academy of Sciences, U.S.A, Copyright 2007.^33^ (C) A methylcyclopropene-modified phospholipid undergoes IEDDA with a tetrazine fluorophore, yielding a fluorescently labelled phospholipid.^37^

Although CuAAC established the feasibility of bioorthogonal lipid visualization, the requirement for copper catalysts limited its application in living systems. The development of SPAAC enabled copper-free labelling under physiological conditions and represented a major transition toward live-cell imaging of lipid dynamics (Fig. 4B).^33^ More recently, inverse electron-demand Diels–Alder (IEDDA) reactions between tetrazines and strained dienophiles have further expanded the capabilities of lipid imaging by combining rapid reaction kinetics with fluorogenic signal activation, allowing high-contrast visualization with minimal background fluorescence (Fig. 4C).^34–38^

A remaining challenge in bioorthogonal lipid imaging is balancing reaction efficiency with probe size. Highly reactive bioorthogonal partners are often relatively bulky and may alter lipid behaviour. Recent development of compact tetrazine-reactive reporters has addressed this limitation by enabling rapid live-cell labelling while maintaining minimal structural perturbation (Fig. 4C).^37^ These advances illustrate that high reactivity and biological fidelity can be achieved simultaneously through careful probe design.

Bioorthogonal probes can also exploit the intrinsic trafficking properties of lipids to achieve organelle-selective visualization.^39^ For example, tetrazine-reactive ceramide analogues have enabled high-resolution imaging of Golgi morphology and ceramide trafficking in living cells through rapid ligation with fluorogenic silicon-rhodamine probes.^39^ Importantly, organelle targeting arises from the native intracellular trafficking behaviour of ceramide rather than from artificial targeting groups, demonstrating that endogenous lipid biology itself can be exploited to guide probe localization.

Beyond modified lipid analogues, bioorthogonal strategies have also enabled the direct detection of endogenous lipid species. Sph and sphinganine [dihydrosphingosine (DHS)] contain a unique 1,2-amino alcohol motif that reacts selectively with salicylaldehyde-derived probes *via* TCL, generating a fluorogenic signal upon covalent labelling (Fig. 5A).^3,40^ Consistent with this mechanism, fluorescence microscopy revealed a Sph concentration-dependent increase in cellular fluorescence, confirming the selective detection of endogenous Sph in HeLa cells (Fig. 5B).^40^ These probes display remarkable selectivity over related metabolites such as sphingosine-1-phosphate (S1P) and have enabled visualization of altered Sph metabolism in disease models, demonstrating that synthetic chemistry can extend beyond tracking exogenous lipid analogues to monitoring native lipid populations.

**Fig. 5:**
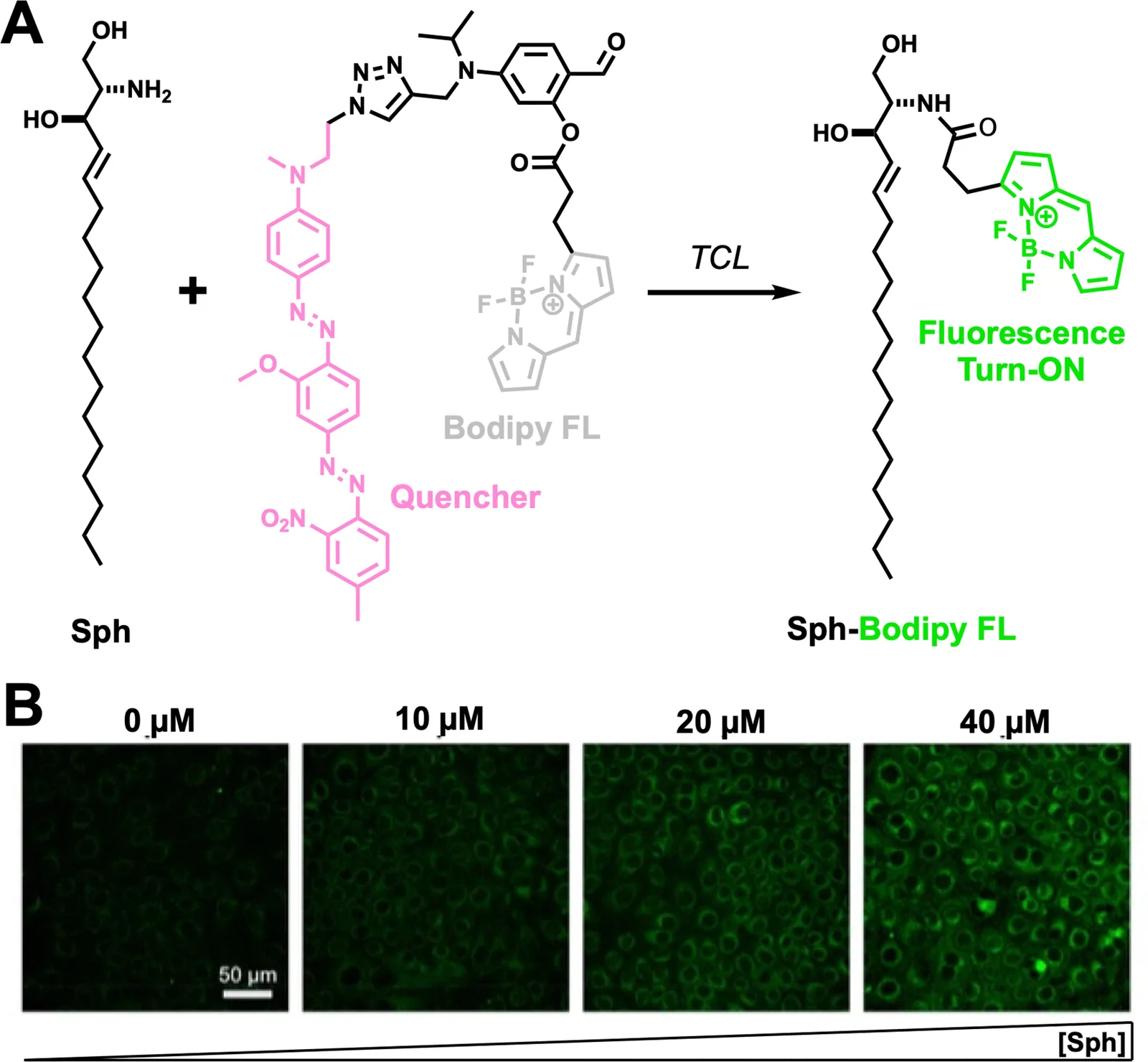
Live-cell detection of sphingosine (Sph) mediated by traceless ceramide ligation (TCL).^40^ (A) Schematic illustration of the fluorogenic reaction between the salicylaldehyde-based probe and Sph. (B) Representative fluorescence microscopy images of HeLa cells incubated with the quencher-BODIPY FL fluorescent probe and subsequently treated with increasing concentrations of Sph (0–40 µM) for 20 h. Figure adapted from ref. 40 with permission from American Chemical Society, copyright 2020.

While bioorthogonal labelling provides detailed information about lipid localization and trafficking, many signalling lipids exert their biological functions through highly localized and transient events that cannot be captured by passive imaging alone.^50–53^ This limitation has motivated the development of photocaged lipids, in which lipid activity is masked by a photolabile protecting group and restored upon irradiation.

Photocaging provides precise temporal control over lipid activation and can also influence spatial distribution through rational design of the protecting group.^50–52^ For example, charged and neutral coumarin-based cages have been used to control whether caged fatty acids remain restricted to the plasma membrane or distribute throughout intracellular membranes.^51^ These approaches demonstrated that the biological outcome of lipid signalling depends not only on lipid identity but also on the precise subcellular location at which lipid activity is initiated. Building on this concept, Wagner *et al.* developed “click cages” containing bioorthogonal alkynes that enable the attachment of organelle-targeting groups, thereby extending photocontrol from the plasma membrane to selected intracellular compartments (Fig. 6).^52^

**Fig. 6:**
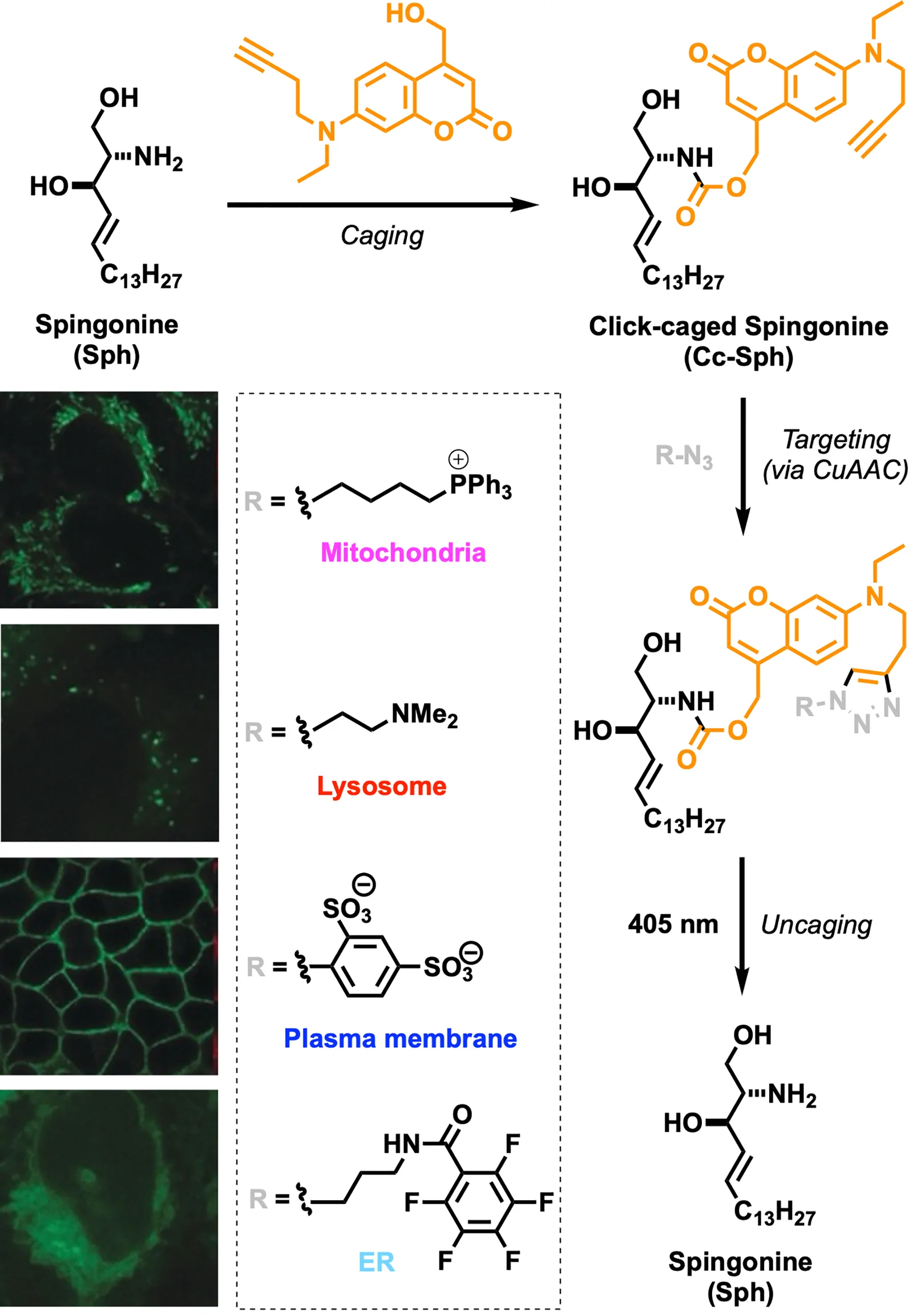
Schematic illustration of organelle-specific uncaging.^52^ Lipid messengers (in this particular case, Sph) are modified with an alkyne-containing photocaging group and subsequently functionalized with organelle-targeting moieties that direct them to specific intracellular compartments (mitochondria, lysosome, plasma membrane, and ER). Upon light irradiation, the active lipid messengers are selectively released (uncaged) within the targeted organelle, minimizing perturbation of messenger levels in neighbouring compartments. Figure adapted from ref. 52 with permission from John Wiley & Sons, copyright 2018.

The combination of photocaging with bioorthogonal chemistry has further expanded this concept by enabling controlled activation of both lipid function and chemical labelling.^53^ Photocaged bioorthogonal reagents allow reactive handles to remain inactive until externally triggered, providing additional control over where and when lipid visualization occurs.^53^ Together, these strategies represent an important transition from simply observing lipid behaviour toward actively manipulating lipid function with defined spatial and temporal precision.

Overall, these technologies have transformed bioorthogonal probes from passive imaging reagents into multifunctional chemical tools capable of coordinating lipid activation, visualization, and functional interrogation with high spatial and temporal precision. More broadly, they exemplify how bioorthogonal chemistry is evolving from a strategy for observing lipid biology to one for actively manipulating lipid function in living systems.^50,53^

### 3.2 Metabolic labelling strategies

Metabolic labelling strategies provide an alternative approach to synthetic lipid probes by exploiting endogenous biosynthetic pathways to generate chemically traceable lipid species inside living systems.^1,41–43^ In this strategy, minimally modified precursors bearing bioorthogonal reporters, most commonly terminal alkynes or azides, are supplied to cells and processed by native metabolic enzymes. Because the chemical reporter is introduced during lipid biosynthesis rather than attached after lipid formation, labelled molecules more closely reflect endogenous lipid production, trafficking, turnover, and metabolism.^1^ Following incorporation, these reporters can be detected through bioorthogonal ligation reactions, enabling fluorescence imaging, affinity enrichment, and quantitative analysis of lipid dynamics in complex biological environments.

Phosphatidylcholine (PC), the most abundant phospholipid in eukaryotic membranes, has served as a major model system for establishing the potential of metabolic lipid labelling. PC is synthesised primarily through the Kennedy pathway, in which choline-containing intermediates are converted into membrane phospholipids through a series of enzymatic reactions with considerable substrate tolerance. This property enabled the development of chemically modified choline analogues capable of entering endogenous phospholipid metabolism while preserving overall lipid homeostasis.^41–43,54–56^

One of the most widely used examples is propargylcholine (ProCho), an alkyne-containing choline analogue that is efficiently incorporated into choline-containing phospholipids, including PCs and sphingomyelins (Fig. 7).^41–43^ Following incorporation, CuAAC-mediated conjugation with fluorescent azides enabled visualization of phospholipid biosynthesis, turnover, and intracellular distribution by fluorescence and electron microscopy.^41–43,54,55^ These studies established a fundamental principle of metabolic lipid imaging: small chemical reporters can provide access to lipid dynamics while avoiding the substantial perturbations associated with direct fluorophore conjugation.^42^

**Fig. 7:**
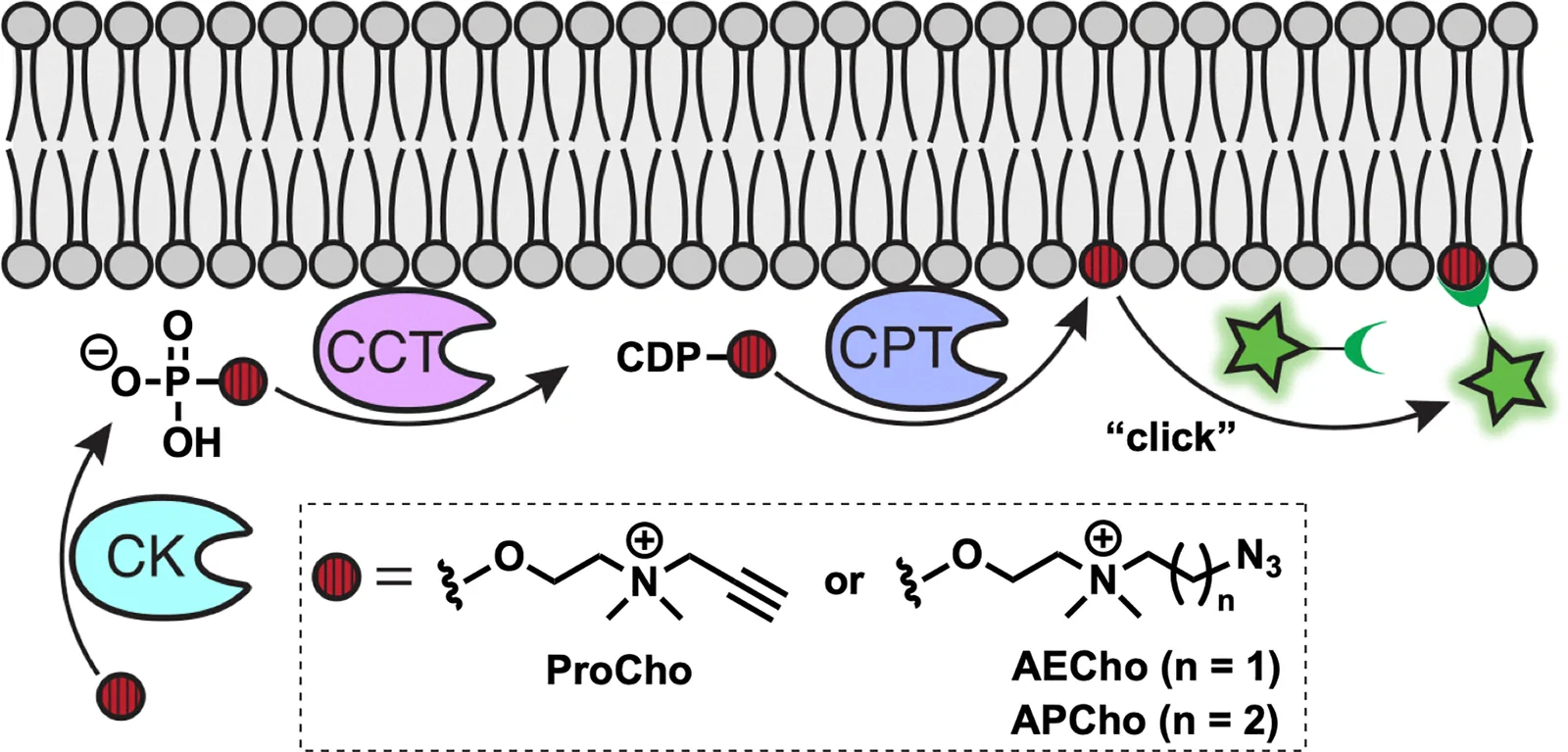
Incorporation of analogues of choline (ProCho, AECho or APCho) *via* the Kennedy pathway of phosphatidylcholine (PC).^41–43^ The resulting tagged lipids can then be selectively labeled *via* click chemistry reactions (CuAAC, SPAAC or Staudinger ligation) using appropriate fluorophore probes, thereby enabling fluorescent imaging [CK: choline kinase, CCT: phosphocholine cytidylyltransferase, CDP: cytidine diphosphate; CPT choline phosphotransferase].

The development of azide-functionalised choline analogues, including azidoethylcholine (AECho) and azidopropylcholine (APCho), further extended this approach to living-cell applications by enabling copper-free ligation reactions such as SPAAC (Fig. 7).^43^ These probes transformed metabolic choline labelling from a fixed-cell imaging strategy into a versatile platform for studying lipid dynamics in real time and demonstrated the importance of matching reporter chemistry with the biological context under investigation.

Beyond mapping lipid distribution, metabolic reporters have revealed fundamental principles of membrane organization. Combining ProCho labelling with ultrastructural imaging enabled direct visualization of newly synthesised choline-containing phospholipids within specific membrane domains, providing insights into lipid asymmetry and membrane remodelling processes that are difficult to resolve through conventional biochemical approaches.^54^ Similarly, metabolic tracing of newly synthesised PC during autophagosome formation demonstrated that lipid incorporation and redistribution are tightly regulated during membrane expansion, revealing a functional role for ATG9-dependent phospholipid scrambling in autophagic membrane biogenesis.^56^

A major conceptual advance was the adaptation of metabolic labelling from monitoring lipid abundance to measuring lipid-producing enzyme activity.^44,45,57–59^ This strategy has been particularly powerful for PA, a signalling lipid whose biological effects depend strongly on its site and mechanism of synthesis. Conventional lipid measurements cannot distinguish between PA pools generated by different enzymes, including phospholipase D (PLD), diacylglycerol kinases, and lysophosphatidic acid acyltransferases.^44^

To overcome this limitation, bioorthogonal alcohol reporters have been exploited as substrates for PLD-mediated transphosphatidylation reactions.^44,45,57^ Instead of simply reporting existing lipid pools, these probes generate labelled phosphatidyl alcohol products specifically at sites of PLD activity, thereby providing a direct readout of lipid synthesis. Early alkyne-based reporters enabled visualization of PLD-generated lipids after CuAAC detection,^44^ whereas the development of azido alcohol probes compatible with SPAAC, including imaging phospholipase D activity with clickable alcohols *via* transphosphatidylation (IMPACT),^45^ extended this approach to living cells (Fig. 8). These studies revealed previously unappreciated spatial heterogeneity in PLD activation and demonstrated that lipid signalling occurs through highly localized metabolic events.

**Fig. 8:**
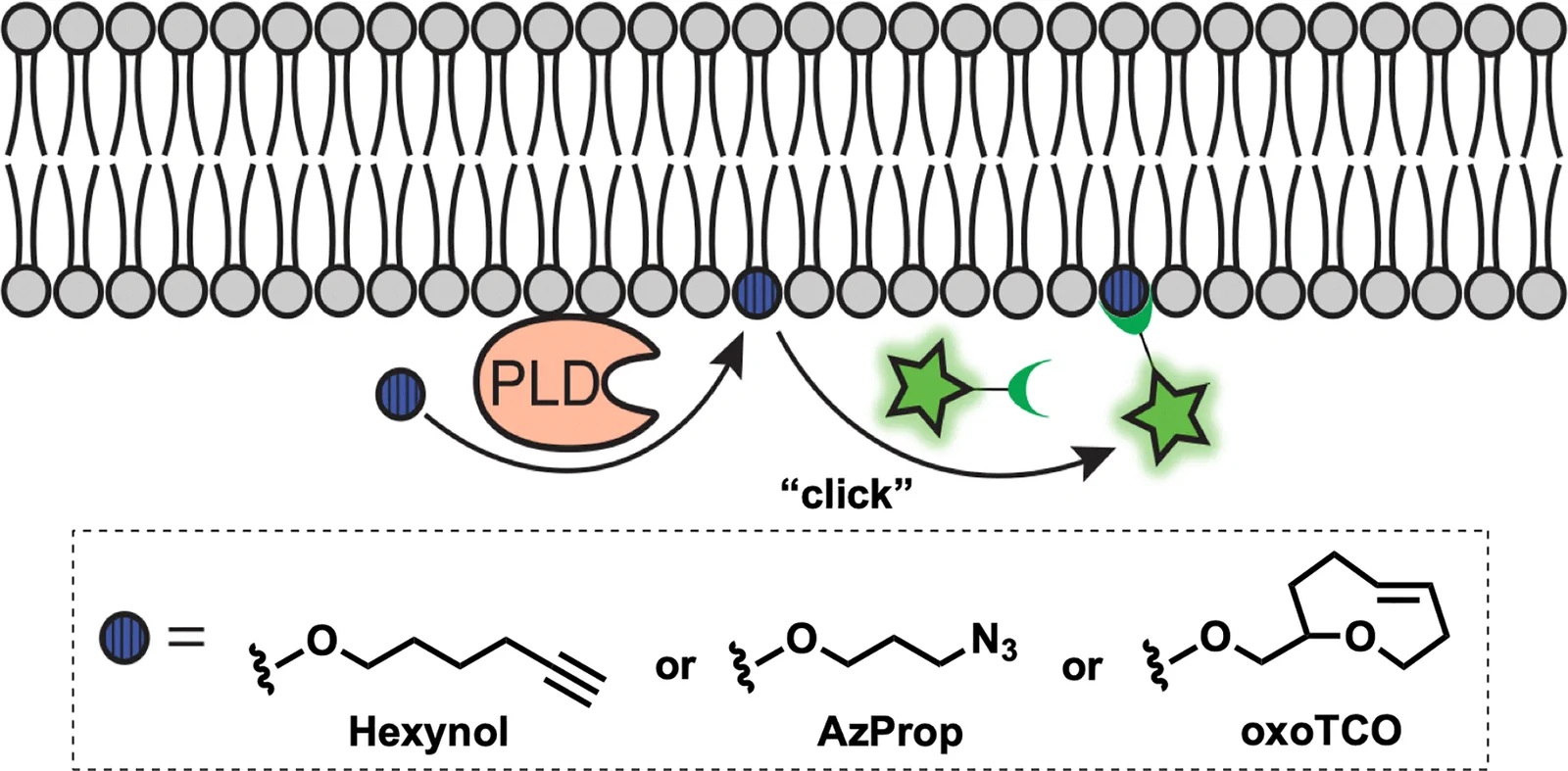
IMPACT labelling employs non-natural primary alcohols to monitor phospholipase D (PLD) activity.^45^ Subsequent fluorophore conjugation through CuAAC, SPAAC, or IEDDA selectively labels phosphatidic acid (PA) pools generated by PLD, enabling their fluorescent visualization [AzProp: 3-azido-1-propanol. oxoTCO: *trans*-5-oxocene].

Improved reaction kinetics further enhanced the temporal resolution of enzyme-activity imaging. *trans*-Cyclooctene (TCO)-containing alcohol reporters combined with tetrazine fluorophores enabled rapid, fluorogenic detection of PLD activity in living cells, allowing visualization of early signalling events following receptor activation (Fig. 8).^58^ Beyond monitoring synthesis, these approaches also uncovered lipid trafficking pathways, demonstrating that PA-derived lipids generated at the plasma membrane can be transferred to the endoplasmic reticulum (ER) through membrane contact sites mediated by lipid-transfer proteins.^59^

The versatility of metabolic labelling is further illustrated by the ability to control metabolic fate through reporter design. Bioorthogonal serine analogues demonstrated that subtle changes in reporter position can redirect labelled phospholipids toward different metabolic pathways, with some reporters tracking phosphatidylserine (PS)-derived lipid networks while others provide greater lipid-class selectivity.^60^ Similar approaches targeting phosphatidylinositol (PI) biosynthesis have enabled visualization of phosphoinositide precursor metabolism, providing new tools to investigate signalling lipid organization within living cells.^46^ More recently, metabolic labelling has also been extended to phosphatidylethanolamine (PE) using clickable ethanolamine analogues, enabling the visualization of PE biosynthesis and trafficking as well as the investigation of PE-dependent cellular processes.^61–63^

Metabolic labelling has also expanded beyond glycerophospholipids to reveal how structural variations within the same lipid class influence biological behaviour.^47,48,64,65^ For instance, clickable diacylglycerol analogues demonstrated that differences in acyl-chain composition can strongly influence intracellular trafficking and interactions with lipid-binding proteins, underscoring that bioorthogonal chemistry can provide functional information beyond simple lipid localization.^48^ These findings highlight an important conceptual advance: bioorthogonal reporters can provide information not only about the localization of lipid species but also about how precise molecular features determines their biological activity.

Despite its versatility, several limitations continue to define the current frontier of metabolic lipid labelling. Because metabolically supplied precursors are often distributed throughout an entire biosynthetic pathway, achieving sufficient spatial specificity to resolve individual lipid pools remains difficult. In addition, fluorophore attachment can still influence lipid behaviour, and relatively slow bioorthogonal reactions may limit temporal resolution in highly dynamic systems.

Emerging strategies are addressing these limitations by combining metabolic incorporation with improved spatial control and more minimally perturbing detection methods. One approach improves spatial resolution by combining metabolic labelling with organelle-targeted clickable fluorophores, in which selective fluorophore localization restricts visualization of labelled lipids to defined subcellular compartments.^4,66^ A recent study from the Budin laboratory exemplifies this strategy by comparing the trafficking of metabolically incorporated alkyne-sphinganine and the non-canonical lipid analogue alkyne-1-deoxysphinganine.^66^ Following metabolic incorporation, both probes were detected by CuAAC using an azide-functionalised Pennsylvania Green fluorophore enriched in ER and Golgi membranes. Comparison with the non-targeted fluorophore AZDye488 picolyl azide revealed that canonical sphingolipids are efficiently transported toward the plasma membrane, whereas 1-deoxysphingolipids accumulate within the ER and ER exit sites. These distinct trafficking patterns linked the accumulation of disease-associated sphingolipid analogues to altered ER membrane properties and impaired secretory trafficking.

Although organelle-targeted fluorophores can substantially improve spatial resolution, their applicability remains restricted to a limited number of intracellular environments and may itself influence lipid distribution. In response, Moore *et al.* developed fluorogen-activating coincidence sensing (FACES), a chemogenetic strategy that combines metabolic labelling with genetically encoded spatial recognition (Fig. 9).^9^ In this approach, azide-labelled lipids generated through metabolic incorporation are detected by fluorogen-activating proteins (FAPs) targeted to specific cellular locations, producing fluorescence only when both components coincide within the same subcellular compartment.^9^ This reversible sensing platform enables quantitative analysis of discrete lipid pools, including PC populations trafficked between the ER and mitochondria by lipid-transfer proteins. Moreover, targeting FAPs to defined membrane leaflets allows FACES to probe the *trans*-bilayer distribution of newly synthesised lipids within the *trans*-Golgi network.^9^ The adaptability of this strategy further extends beyond lipid biology, providing a general framework for visualizing other classes of metabolically labelled biomolecules.

**Fig. 9:**
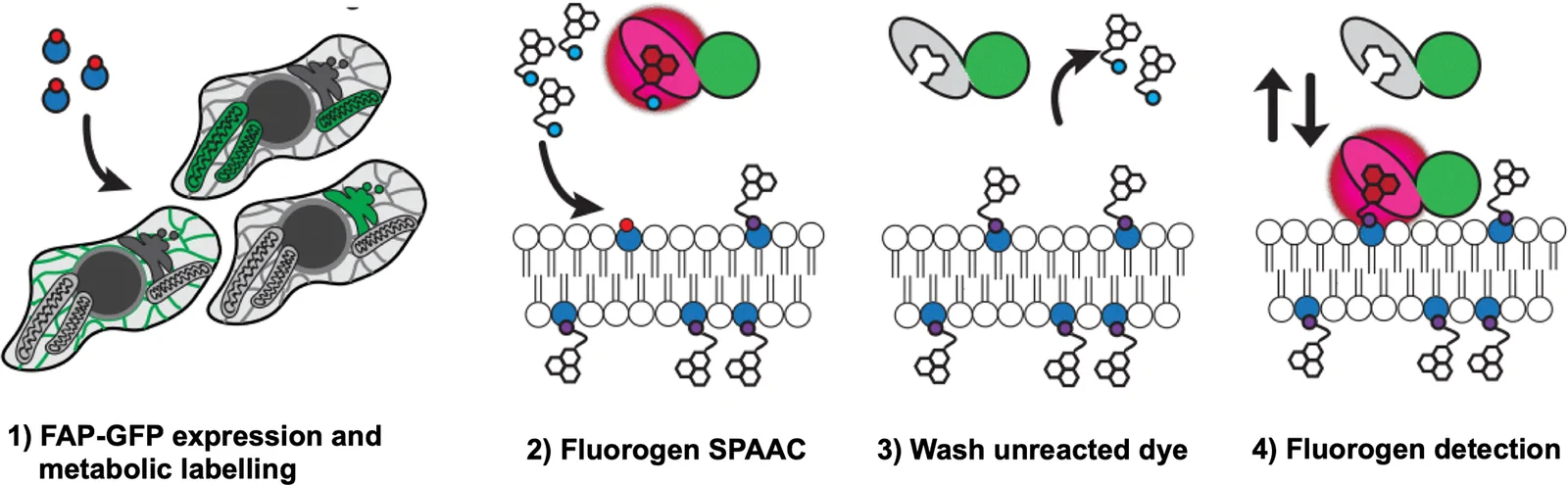
Schematic overview of the FACES method.^9^ Cells expressing a FAP–GFP fusion protein localized to a target organelle are metabolically labelled with a bioorthogonal metabolite (1), such as azidocholine (N_3_–Cho). The incorporated metabolite is then conjugated to a fluorogen-DBCO probe *via* a SPAAC reaction, allowing transient binding of the free fluorogen to the FAP and generating far-red fluorescence (2). After labelling, unbound dye is removed by washing, leaving only the fluorogen-FAP complexes (3). Finally, fluorescence imaging is performed to detect the labeled biomolecule pool, while GFP fluorescence is used to normalize the fluorogen signal according to FAP sensor expression levels (4). Figure adapted from ref. 9 with permission from Springer Nature, copyright 2026.

An alternative approach addresses the spatial limitations of fluorescence imaging from a physical rather than a biochemical perspective. In lipid expansion microscopy, metabolically labelled lipids are covalently anchored to a swellable hydrogel through click chemistry prior to sample expansion. Subsequent isotropic expansion increases the physical separation between labelled molecules, enabling nanoscale visualization of membrane structures using conventional diffraction-limited microscopes.^49^ By overcoming optical resolution constraints through physical magnification, expansion microscopy provides access to lipid organization at spatial scales inaccessible to standard fluorescence imaging.

Together, these advances are progressively overcoming the major spatial limitations of conventional metabolic labelling and enabling the visualization of lipid organization at the level of individual organelles, membrane leaflets, and sub-diffraction structures. Nevertheless, the perturbation associated with fluorophore-based detection continues to motivate the development of smaller and less invasive labelling strategies. This need has stimulated growing interest in vibrational imaging approaches, which employ minimal Raman-active tags and eliminate the requirement for secondary labelling reactions, as discussed in the following section.

### 3.3 Stimulated Raman scattering (SRS) spectroscopy

Fluorescence microscopy combined with bioorthogonal labelling has become one of the most widely used approaches for visualizing lipids in living systems because of its high sensitivity, versatility, and broad accessibility. However, fluorescence-based methods also present inherent limitations. The relatively large size of fluorophores can perturb lipid behaviour, prolonged illumination may cause photobleaching and phototoxicity, and the broad emission spectra of fluorescent dyes restrict the number of molecular species that can be imaged simultaneously.^67^ These challenges have driven the development of alternative imaging modalities capable of providing chemically specific information with minimal molecular perturbation.^67–70^ Among these, Raman-based imaging has emerged as a particularly powerful approach.^67–70^ Rather than detecting fluorescence emission, Raman spectroscopy probes the vibrational modes of chemical bonds, generating molecular fingerprints that enable direct chemical identification. Moreover, biomolecules can be labelled with extremely small vibrational reporters, commonly referred to as Raman tags, which introduce substantially less structural perturbation than conventional fluorophores.^67^

Raman tags are small chemical groups whose characteristic vibrational frequencies fall within the cell-silent region of the Raman spectrum (approximately 1800–2800 cm^−1^), where endogenous biomolecules generate little or no signal. The most widely used reporters include terminal alkynes (C≡C), conjugated diynes, nitriles (C≡N), and carbon–deuterium (C–D) bonds. Because these modifications introduce only minimal structural perturbation while generating intense, well-resolved vibrational signals, they are particularly well suited for imaging lipid metabolism while preserving native lipid behaviour.^67^

Despite these advantages, spontaneous Raman scattering is intrinsically insensitive because only a minute fraction of incident photons undergo inelastic scattering. Stimulated Raman scattering (SRS) microscopy overcomes this limitation by coherently amplifying selected vibrational modes, enabling rapid, background-free imaging with high chemical specificity, optical sectioning capability, low phototoxicity, and imaging speeds compatible with dynamic studies in living cells.^68,69^ Continuous improvements in instrumentation have further enhanced the sensitivity, temporal resolution, and spatial resolution of SRS microscopy.^70^

These characteristics make SRS particularly well suited for investigating lipid metabolism in living systems. Small alkyne- or deuterium-labelled metabolic precursors are readily incorporated into endogenous biosynthetic pathways, enabling direct visualization of the *de novo* synthesis of phospholipids, neutral lipids, and other biomolecules in living cells and whole organisms.^68^ Terminal alkynes are especially attractive because they generate intense, spectrally isolated Raman signals within the cell-silent region, while the distinct vibrational frequencies of different Raman reporters facilitate multiplexed imaging without the spectral overlap that limits fluorescence-based approaches.^68^

Beyond monitoring lipid biosynthesis, SRS has provided important insights into membrane organization and biophysical properties.^71^ For instance, imaging the C–D signal of deuterated palmitate revealed the formation of micron-sized, solid-like membrane domains within the ER during saturated fatty-acid metabolism, whereas co-administration of oleate prevented their formation.^71^ These previously unrecognised membrane structures established a direct link between fatty-acid saturation, membrane order, and lipotoxicity, illustrating how chemically selective imaging can reveal emergent membrane behaviour.

Improvements in spectral resolution have further expanded the analytical capabilities of SRS. Hyperspectral SRS can discriminate lipid species differing in acyl-chain length or degree of unsaturation directly within intact cells, opening opportunities for chemically resolved lipid phenotyping in metabolic disorders and cancer.^70^

The applicability of Raman-tag imaging also extends beyond cultured cells (Fig. 10). Administration of heavy water (D_2_O) results in the incorporation of deuterium into newly synthesised lipids, enabling visualization of *de novo* lipogenesis throughout living animals and identifying metabolically active tissues such as tumours (Fig. 10).^72^ Likewise, alkyne- and diyne-labelled sterol analogues have enabled direct imaging of cholesterol transport and storage, revealing abnormal intracellular cholesterol trafficking in disease models such as Niemann-Pick type C while preserving the narrow vibrational signatures required for multiplexed detection.^73,74^

**Fig. 10:**
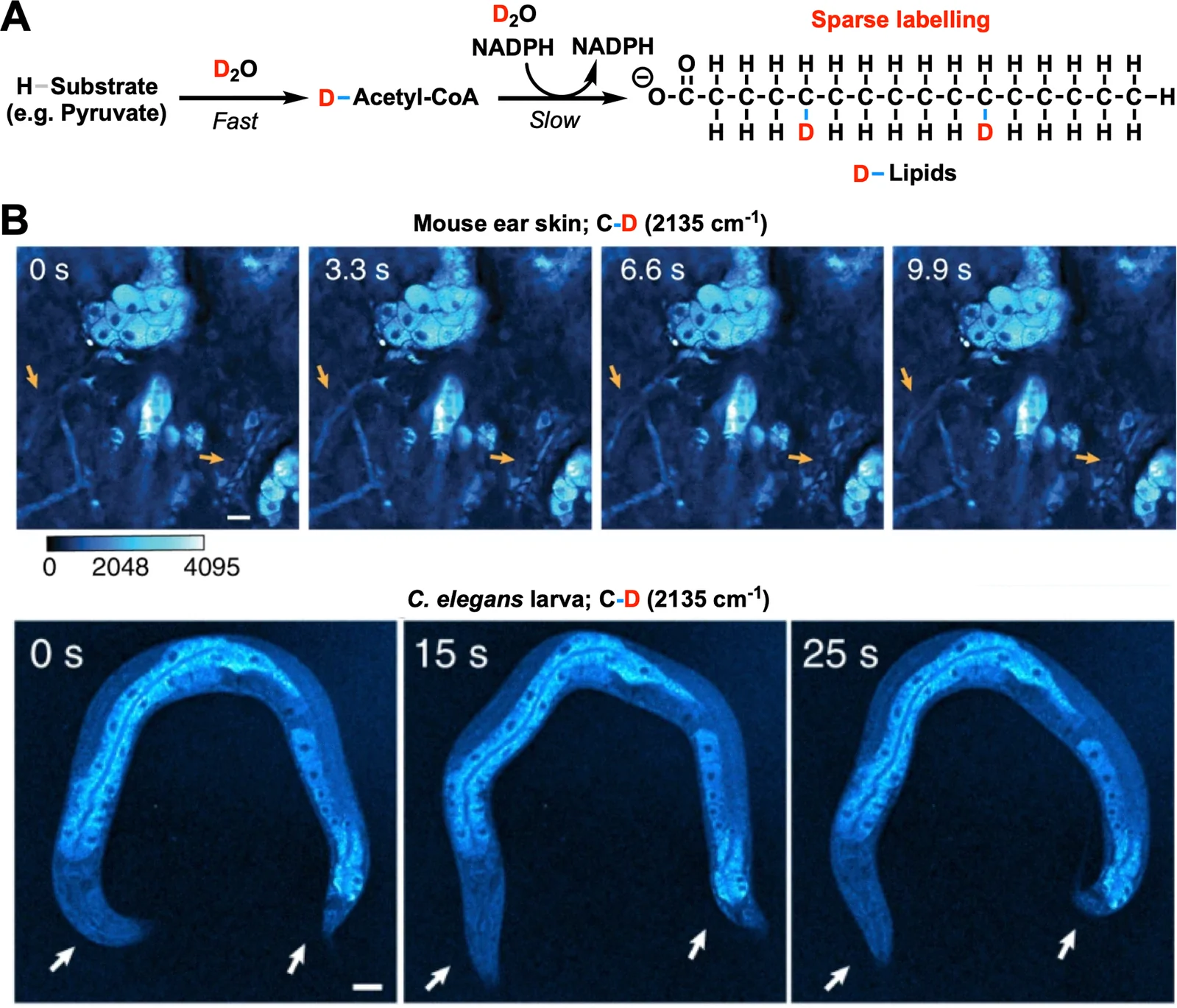
SRS imaging of *de novo* lipid synthesis by metabolic D_2_O labelling in living animals.^72^ (A) Deuterium from D_2_O is irreversibly incorporated into C–D bonds during lipid biosynthesis. Under sparse-labelling conditions, deuterium is randomly distributed throughout lipid molecules; the illustrated structure represents one possible labelling pattern. (B) Representative live SRS images of sebaceous glands in mice administered 25% D_2_O for 9 days (top) and fourth-stage *C. elegans* larvae exposed to 20% D_2_O for 4 h (bottom). Blood flow in mice (orange arrows) and body movement in *C. elegans* (white arrows) confirm imaging of living animals. C–D signals are shown using a cyan hot lookup table (LUT). Scale bars: 20 µm. Figure adapted from ref. 72 with permission from Springer Nature, copyright 2018.

Nevertheless, although SRS provides minimally perturbing visualization of lipid metabolism with exceptional chemical specificity, complementary bioorthogonal approaches remain necessary to identify the molecular interactions and protein partners through which lipids exert their biological functions. The following section therefore focuses on bioorthogonal strategies that move beyond lipid imaging to directly interrogate lipid–protein interactions.

## 4. Protein–lipid interactions

A large proportion of lipid function is mediated through interactions with proteins, either by the covalent attachment of lipid moieties to proteins or through non-covalent recognition of membrane lipids by lipid-binding domains.^10,30,75^ These interactions regulate virtually every aspect of membrane biology, including protein trafficking, signal transduction, membrane remodelling, organelle identity, and cell communication.^10,30,75^ Their characterization has historically been challenging because lipid modifications are chemically diverse, highly dynamic, and generally incompatible with conventional labelling strategies. Bioorthogonal chemistry has fundamentally transformed this field by enabling the selective visualization, enrichment, and proteomic analysis of lipid-modified proteins in complex biological environments.^10,30,75,76^ This section focuses on the major bioorthogonal approaches that have made post-translational protein lipidation experimentally accessible and have provided new insights into lipid-mediated protein regulation.

### 4.1 Post-translational lipidation of proteins

In addition to labelling free lipid pools, metabolically incorporated bioorthogonal reporters can trace lipids that become covalently attached to proteins. Protein lipidation constitutes a major class of post-translational modifications (PTMs) in which hydrophobic lipid moieties regulate membrane association, subcellular localization, intracellular trafficking, protein stability, and protein–protein interactions.^30,75^ Because many lipidation events are dynamic and tightly regulated, they provide an important mechanism for controlling protein function in space and time.

The major lipid PTMs in eukaryotic cells include S-palmitoylation, in which palmitate is attached to cysteine residues through a thioester bond; *N*-myristoylation, where myristate is linked to an N-terminal glycine through an amide bond; and S-prenylation, in which farnesyl or geranylgeranyl isoprenoids are covalently attached to C-terminal cysteine residues through stable thioether linkages. Less common lipid modifications include N- and O-acylation, Nε-lysine acylation, cholesterylation, and glycosylphosphatidylinositol (GPI) anchoring.^30,75^ Among these PTMs, reversibility represents an important functional distinction. Whereas S-palmitoylation undergoes rapid cycles of acylation and deacylation, N-myristoylation and S-prenylation are generally considered irreversible under physiological conditions, allowing these modifications to fulfil distinct biological roles.^30,77^

Historically, protein lipidation was as difficult to investigate as free lipid metabolism. Radioactively labelled fatty acids and isoprenoid precursors required lengthy autoradiographic exposure times and involved significant safety concerns, whereas directly fluorescent lipid analogues were often too bulky to be efficiently recognised by endogenous lipidation enzymes or altered the properties of the modified proteins.^75^ The introduction of metabolic chemical reporters largely overcame these limitations and now underpins most modern approaches for studying protein lipidation.^75,78–83^

The principal bioorthogonal ligation reactions used to detect lipid-modified proteins include the Staudinger ligation, CuAAC, and SPAAC.^75^ Early studies relied on azido-fatty acids coupled to phosphine probes through the Staudinger ligation (Fig. 11A).^78^ However, subsequent comparisons demonstrated that alkynyl fatty-acid reporters combined with CuAAC provide substantially higher sensitivity and lower background, particularly for in-gel fluorescence detection (Fig. 11B).^79,80^ These advances replaced radioactive detection with rapid, robust, and non-radioactive workflows that dramatically expanded the accessibility of protein lipidation analysis.^30,75^

**Fig. 11:**
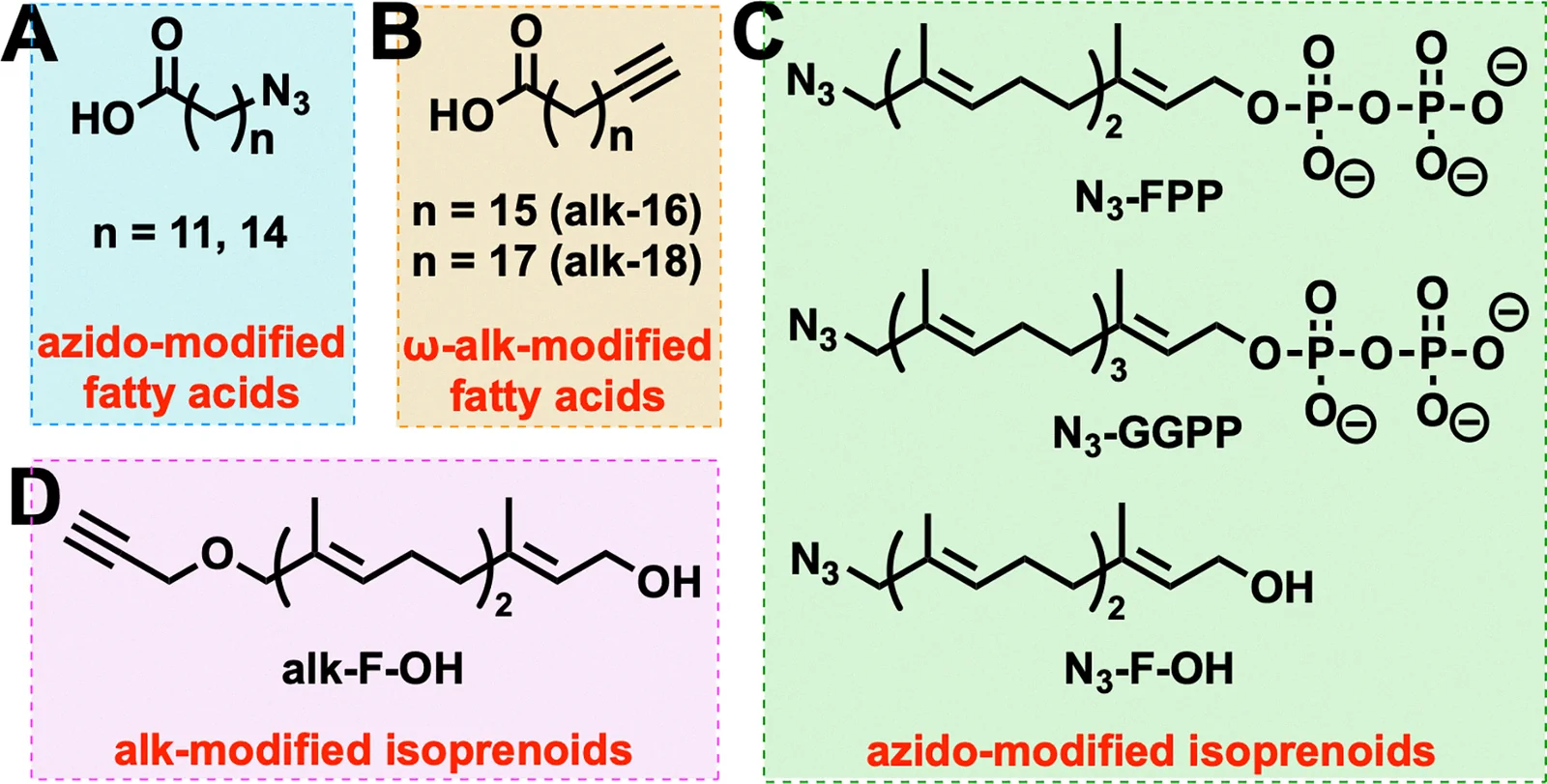
Chemical probes for investigating protein post-translational lipidation. (A) ω-azido-fatty acids enable rapid detection of protein fatty acylation through Staudinger ligation.^78^ (B) Terminal alkyne-containing fatty acids facilitate the analysis of protein lipidation *via* CuAAC.^80^ (C) Azide-functionalised isoprenoids, available as diphosphate (top) or alcohol (bottom) derivatives, enable the identification of protein prenylation sites.^85^ (D) Alkyne-functionalised isoprenoids are used to identify protein prenylation sites across the proteome.^86^

Among the available metabolic reporters, ω-functionalised fatty acid analogues have emerged as the most versatile probes for studying cytosolic fatty-acyl modifications.^75,76,78–82^ The best-characterised examples are the alkynyl palmitate analogue alk-16 and the corresponding stearate analogue alk-18, which are converted intracellularly into their respective acyl-CoA derivatives and efficiently recognised by endogenous acyltransferases (Fig. 11B).^13,30,80^

*N*-Myristoylation is catalysed by *N*-myristoyltransferases (NMTs), either co-translationally following removal of the initiator methionine or post-translationally after caspase-mediated cleavage during apoptosis.^30,77^ Analogous alkynyl–myristate reporters, together with selective NMT inhibitors, have enabled comprehensive mapping of the human N-myristoylated proteome while distinguishing co-translational from post-translational substrates.^83^

Although these metabolic reporters faithfully recapitulate endogenous lipid metabolism, they also illustrate an important limitation of precursor-based labelling: a single fatty acid analogue does not discriminate between different types of lipid modification. For instance, alk-16 is incorporated not only into S-palmitoylated proteins but also in N- and O-acylated proteins, as well as GPI-anchored proteins.^76^ Consequently, metabolic incorporation alone cannot identify either the modified amino acid or the nature of the covalent linkage. This limitation is commonly addressed by exploiting the distinct chemical stability of different lipid linkages. The thioester bond of S-palmitoylation is selectively cleaved by hydroxylamine, whereas the amide bond of N-myristoylation and the lipid linkage within GPI anchors remain resistant. Hydroxylamine sensitivity therefore provides a simple and effective means of distinguishing S-acylated proteins from other classes of lipid-modified proteins.^30,76^

The reversibility of S-palmitoylation is itself a biological feature that metabolic reporters have made experimentally accessible. Pulse-chase experiments using alk-16 enabled dynamic cycles of acylation and deacylation to be monitored directly in living cells, revealing turnover times ranging from seconds to hours.^75,77,82^ Because palmitoyl-CoA serves both as a central metabolic intermediate and as the lipid donor for protein acylation, S-palmitoylation has emerged as a key regulatory node linking lipid metabolism to membrane trafficking and signal transduction.^77^

Bioorthogonal reporters have also uncovered important unresolved questions. For instance, azido-palmitate labelling revealed a distinct population of S-acylated proteins within the mitochondrial matrix,^84^ yet whether these modifications arise through enzymatic catalysis or spontaneous acyl transfer driven by the high intramitochondrial acyl-CoA concentration remains unclear.^77^ Such findings illustrate that metabolic reporters do more than catalogue lipidated proteins; they generate new mechanistic hypotheses that earlier methodologies could not readily address.

Protein prenylation can likewise be monitored using azido- or alkynyl-functionalised analogues of the isoprenoid donors farnesyl diphosphate (FPP) and geranylgeranyl diphosphate (GGPP) (Fig. 11C, top).^30,75,85,86^ Because prenyltransferases display relatively broad substrate tolerance, these reporters are efficiently incorporated into target proteins. Farnesyltransferase (FTase) and geranylgeranyltransferase-I (GGTase-I) modify proteins containing a C-terminal CAAX motif, whereas RabGGTase (or GGTase-II) catalyzes the addition of geranylgeranyl groups to paired C-terminal cysteine residues in Rab GTPases.^30,75^ The first bioorthogonal prenylation reporter, an azido-farnesol analogue detected through the Staudinger ligation (Fig. 11C, bottom),^85^ was subsequently superseded by more sensitive alkynyl isoprenoid analogues compatible with CuAAC (Fig. 11D).^86^

Compared with fatty acid reporters, metabolic labelling of the prenylome presents an additional challenge because efficient incorporation generally requires depletion of endogenous isoprenoid pools through statin treatment.^30,75^ Nevertheless, bioorthogonal prenylation reporters have substantially expanded the known prenylome, uncovered previously unrecognised prenylated proteins, and contributed to the identification of the fourth prenyltransferase, GGTase-III.^30,75^ Similar strategies have also been extended to Hedgehog cholesterylation and GPI anchoring using azide-functionalised sterol reporters.^30,75^

Together, bioorthogonal metabolic reporters have transformed the study of protein lipidation from the analysis of individual substrates into a quantitative, proteome-wide discipline that is increasingly capable of providing site-specific information.^81^

A recurring theme emerging from these studies is the complementarity between metabolic labelling and the hydroxylamine-based enrichment methods such as acyl–biotin exchange (ABE) and acyl–resin-assisted capture (acyl–RAC).^87,88^ Whereas hydroxylamine-based methods recover all thioester-linked proteins irrespective of the attached lipid, metabolic reporters selectively trace proteins incorporating a defined lipid analogue. Conversely, metabolic reporters may undergo β-oxidation or metabolic remodelling before incorporation. Consequently, proteins identified independently by both approaches generally constitute the highest-confidence S-acylation datasets.

While proteome-wide identification represented a major advance, understanding the biological consequences of lipidation ultimately requires defining both the modified residue and the identity of the attached lipid. Thinon *et al.* combined alk-16 labelling with hydroxylamine discrimination and direct tagging of liberated cysteine residues, enabling simultaneous enrichment of S-fatty-acylated proteins and mapping of their modification sites (Fig. 12).^76^ Applied to the interferon-induced transmembrane protein IFITM3, this strategy identified the principal sites of S-palmitoylation and established palmitate as the predominant attached fatty acid, providing one of the first direct links between a defined lipidation site and protein function.^75,76,89^

**Fig. 12:**
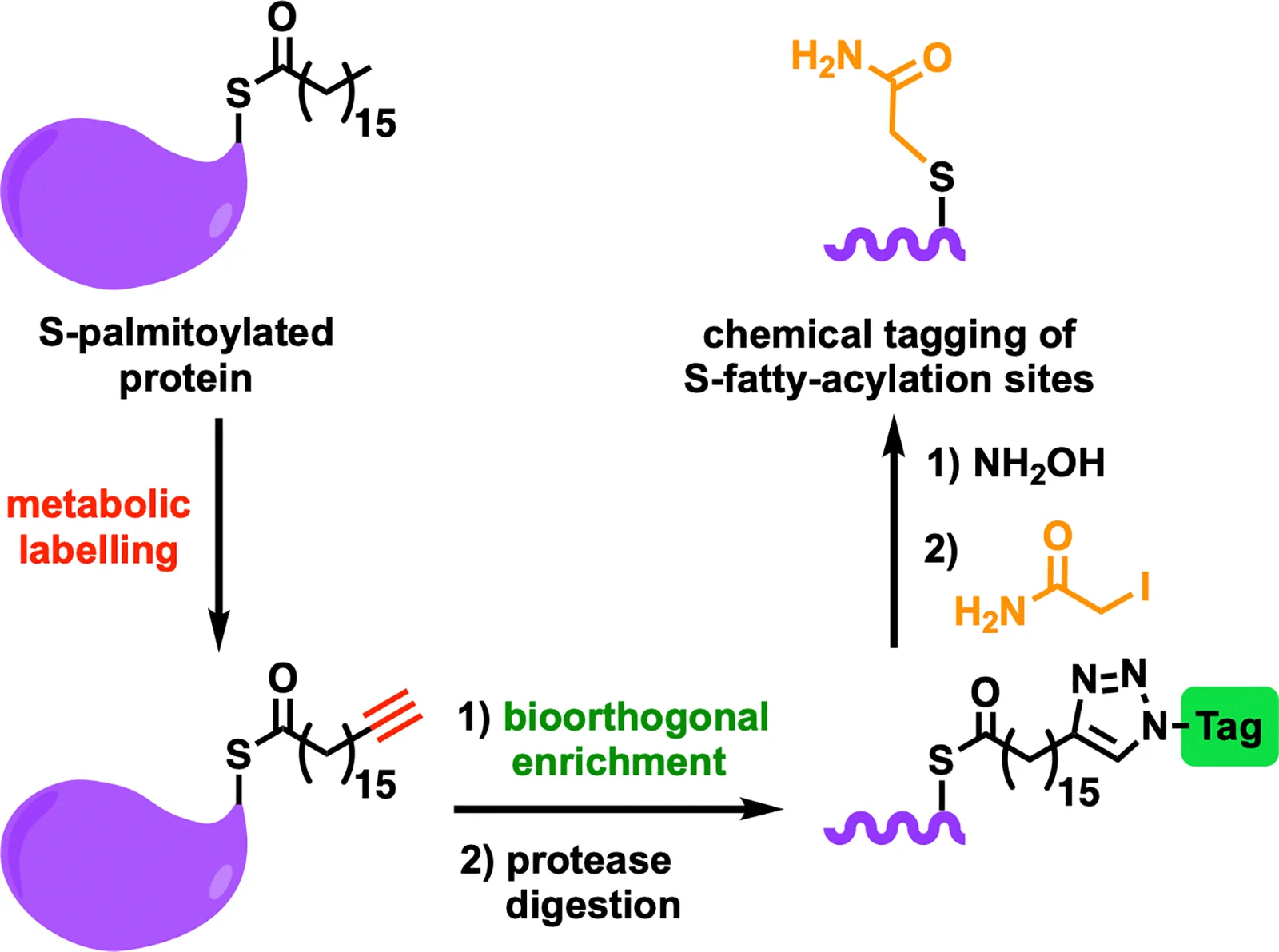
Workflow integrating fatty acid chemical reporter labelling with hydroxylamine-mediated enrichment and direct tagging of modified cysteine residues to selectively identify sites of S-fatty acylation.^76^

Subsequent methodological advances in quantitative chemoproteomics have greatly expanded site-resolved lipidomics. Cleavable capture reagents incorporating affinity handles and protease-cleavable linkers improve recovery and mass spectrometric analysis of hydrophobic lipidated peptides,^30^ while complementary mass-tagging strategies enable quantitative analysis of endogenous, site-specific S-fatty-acylation.^90^ More recent methodologies recover intact S-acylated peptides, allowing modification sites, fatty acid identity, and turnover kinetics to be determined simultaneously.^91^ Combined with stable isotope labelling by amino acids in cell culture (SILAC), tandem mass tag (TMT), and pulse-chase experimental designs, these approaches distinguish constitutively modified proteins from rapidly cycling substrates and establish protein lipidation as a highly dynamic signalling mechanism.^92,93^

Future progress will depend on overcoming several remaining challenges. Current metabolic reporters generally label entire classes of lipidated proteins rather than individual substrates, highlighting the need for enzyme-selective and site-specific probes. In addition, reporter groups introduced following bioorthogonal ligation may perturb membrane behaviour, and the application of these methodologies in whole organisms remains considerably less mature than in cultured cells. Continued advances in probe design, bioorthogonal chemistry, and quantitative proteomics are therefore expected to move the field from proteome-wide cataloguing towards a comprehensive, protein-resolved, and ultimately *in vivo* understanding of lipid PTMs.

### 4.2 Bioorthogonal lipid probes with photo-crosslinking

Lipids regulate protein function not only through covalent modifications but also through noncovalent interactions that control processes such as enzyme activity, receptor signalling, membrane trafficking, and assembly of supramolecular protein complexes. Compared with covalent lipidation, however, noncovalent lipid–protein interactions have proven considerably more difficult to characterize. Lipid-binding sites are rarely predictable from primary sequence, and many biologically relevant interactions are transient, weak, or dependent on the native membrane environment, leading to their disruption during cell lysis and biochemical purification.^10,94^ Conventional approaches, including affinity chromatography, calorimetry, thermal-shift assays, structural biology, and molecular docking, therefore often provide only a partial view of lipid–protein recognition and typically rely on purified proteins removed from their physiological context.^10^

Bioorthogonal lipid probes incorporating photoactivatable crosslinkers provide an effective solution to these limitations. Upon irradiation, transient noncovalent interactions are converted into stable covalent adducts that withstand cell lysis and downstream sample processing, enabling interacting proteins to be enriched, identified, and, in many cases, mapped at amino acid resolution.^10,94,95^ Because crosslinking occurs within intact membranes, these probes preserve the native lipid environment in which protein–lipid interactions take place, providing access to physiologically relevant membrane interactomes that are difficult to capture using conventional biochemical approaches.^10,94,95^

Most photoaffinity probes share a modular architecture comprising three functional elements: (i) a lipid scaffold that preserves the molecular recognition properties of the parent lipid, (ii) a photoactivatable group that generates a reactive intermediate upon irradiation and mediates covalent crosslinking; and (iii) a minimally perturbing bioorthogonal handle, typically an azide or alkyne, that enables subsequent conjugation to fluorescent or affinity reporters through bioorthogonal ligation (Fig. 13).^10^ This modular design separates biological interrogation from probe detection, reducing perturbation during the experiment while providing flexibility for downstream imaging and proteomic analyses.

**Fig. 13:**
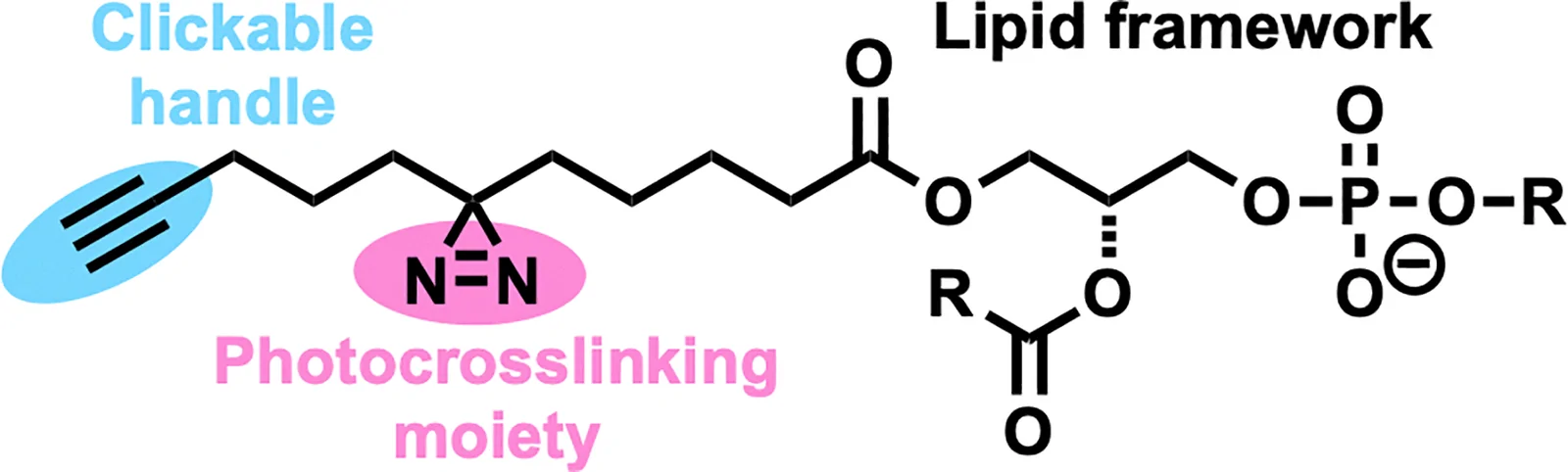
Schematic representation of a photocrosslinking probe.

The choice of the photoactivatable group is a critical determinant of probe performance. Aryl azides, benzophenones, and diazirines generate reactive intermediates with distinct lifetimes and labelling selectivities. Although benzophenones provide efficient crosslinking through long-lived excited states, their larger size can influence lipid recognition and increase nonspecific labelling. Aryl azides often display lower efficiency due to competing side reactions. Consequently, alkyl diazirines have become the preferred crosslinking groups for lipid probes because they combine minimal steric perturbation with efficient carbene formation and activation wavelengths compatible with biological samples.^10^

The position of the photoreactive group within the lipid scaffold is equally important. Incorporation within the hydrophobic acyl chain favours capture of transmembrane protein surfaces embedded within the bilayer, whereas placement near the lipid headgroup preferentially labels peripheral membrane proteins and interactions occurring at the membrane–water interface.^94^ Accordingly, probe development generally requires structure–activity optimization to preserve the charge, polarity, stereochemistry, and biological behaviour of the native lipid.^10,96^ Studies using trifunctional fatty acid probes have shown that even small changes in diazirine position can substantially alter cellular uptake, metabolism, and protein labelling profiles.^97^

Early applications of photoactivatable lipid probes focused on phospholipid–protein interactions at membrane interfaces. Gubbens *et al.* developed an aryl azide-functionalised PE analogue capable of incorporation into membranes, enabling lipid–protein adducts to be identified by mass spectrometry rather than radioactive detection (Fig. 14A).^94^ This approach demonstrated that membrane lipid interactions could be trapped in native bilayers and analysed at the proteome level.^94^

**Fig. 14:**
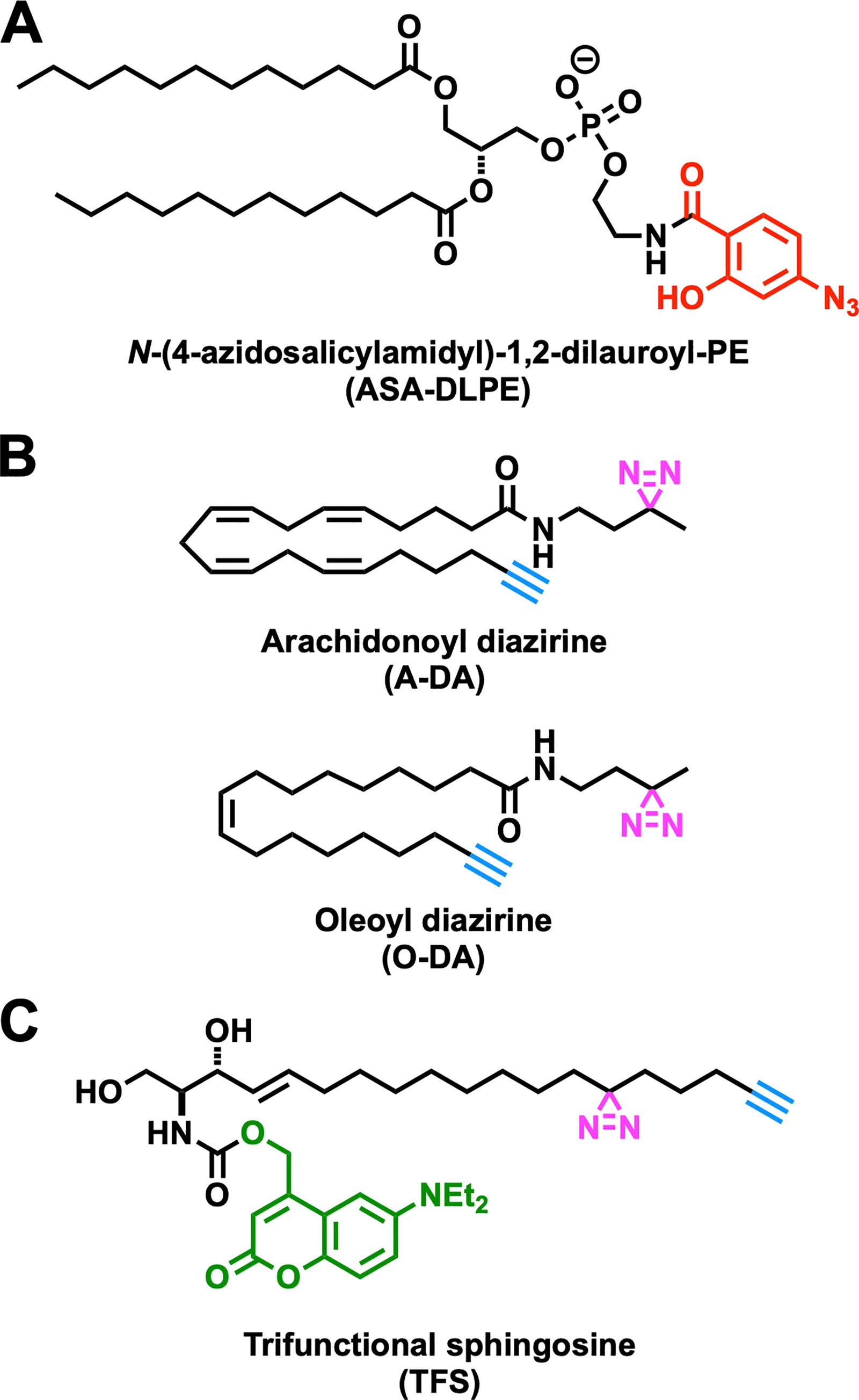
Photoaffinity lipid probes used to investigate lipid–protein interactions and lipid metabolism. (A) Phospholipid analogue containing an aryl azide-based photoactivatable group.^94^ (B) Bifunctional lipids featuring both a photocrosslinkable diazirine group and a terminal alkyne handle.^99^ (C) Trifunctional sphingosine (TFS) bearing a photocaged group, a photocrosslinkable diazirine and a terminal alkyne.^100^

A major advance came with the incorporation of bioorthogonal reporter handles into photoaffinity lipids. This modification transformed photo-crosslinking probes from qualitative interaction traps into multifunctional tools for selective enrichment and quantitative analysis. Following irradiation, the bioorthogonal handle can be derivatised with fluorescent reporters for imaging or with affinity tags such as biotin for enrichment and liquid chromatography-tandem mass spectrometry (LC-MS/MS) identification.^10^ Because reporter installation occurs after crosslinking, the native properties of the lipid probe are preserved during the biological experiment.

This modular strategy has since been extended to a wide range of lipid classes, including sterols, sphingolipids, phosphoinositides, and fatty acids, as well as less extensively characterised lipids such as *N*-acylphosphatidylethanolamines (NAPEs).^10,98^ In the case of NAPEs, photoaffinity proteomics revealed an unexpected role for these lipids in regulating cellular lactate homeostasis, illustrating how unbiased interaction mapping can uncover previously unknown lipid functions.^98^ Collectively, these approaches have generated the first proteome-wide maps of lipid–protein interactions in mammalian cells and greatly expanded the range of lipid signalling pathways accessible to chemical biology.

The power of bifunctional photoaffinity probes is further enhanced when combined with quantitative chemoproteomics. Cravatt and co-workers developed arachidonoyl- and oleoyl-derived probes containing both a diazirine crosslinker and an alkyne reporter to generate the first proteome-wide maps of lipid-binding proteins and their ligandability in living cells (Fig. 14B).^99^ This strategy relies on competitive binding: occupation of lipid-binding sites by exogenous molecules reduces probe crosslinking in proportion to binding affinity, allowing cellular targets of bioactive compounds to be identified without chemically modifying the compounds themselves.

Competitive photoaffinity profiling has become a powerful approach for target identification and mechanism-of-action studies.^10,95,99^ For instance, this strategy identified the prostaglandin reductase PTGR2 as a target of the hop-derived antidiabetic natural product KDT501 and the sphingosine-1-phosphate lyase (S1PL) as a target of the antifungal drug miconazole.^95^ Beyond target discovery, tandem mass spectrometry analysis of crosslinked peptides can provide information about ligand-binding regions, generating structural hypotheses even for proteins lacking established lipid-binding domains.^95^ Together, these approaches have positioned lipid-based photoaffinity probes as versatile platforms for target identification, ligandability assessment, and therapeutic discovery.

A complementary development has focused on integrating photo-crosslinking with precise spatiotemporal control. Conventional photoaffinity probes identify interaction partners but provide limited information about where and when lipid signalling events occur. To address this limitation, the Schultz group developed trifunctional probes integrating a photocaged lipid, a diazirine crosslinker, and a bioorthogonal reporter handle (Fig. 14C).^100^ This design combines the temporal precision afforded by photocaged lipids with the proteomic capabilities of bifunctional photoaffinity probes.

A photocaged PI probe illustrates the potential of this strategy.^96^ A photoremovable protecting group enables intracellular delivery of the lipid, while sequential irradiation allows independent control over lipid release and protein capture. Following uncaging, the diazirine group can be activated to trap nearby interacting proteins, and the alkyne handle enables fluorescence imaging or affinity enrichment for proteomic analysis.^96^ Application of this approach revealed that PI initially accumulates on intracellular membranes before redistributing to the plasma membrane and identified transfer proteins PITPα and PITPβ among its principal interaction partners.^96^

Despite these advances, several technical challenges continue to limit the broader application of photoaffinity lipid probes. Multifunctional photoaffinity probes are often synthetically challenging because lipid scaffolds provide few positions that can be modified without altering biological activity.^10^ In addition, photo-crosslinking efficiency is intrinsically limited. Studies using defined protein–lipid complexes have shown that only a fraction of photoactivated diazirines generate productive covalent adducts, while competing reactions produce inactive by-products.^101^ Consequently, careful experimental controls, including nonirradiated samples, inactive probe analogues, and competition experiments with native lipids, remain essential for distinguishing specific interactors from background binding.^10,95^

Current photoaffinity approaches still cover only a limited fraction of the cellular lipidome. Many important lipid classes, including signalling lysophospholipids and oxidised lipids generated during ferroptosis, remain largely inaccessible. Moreover, most studies have been performed in cultured cells, with comparatively few applications in tissues or whole organisms. Expanding the chemical diversity of photoaffinity probes while improving their applicability in increasingly native physiological environments represents a major challenge and opportunity for the next generation of bioorthogonal lipid chemical biology.^10^

## 5. Therapeutic applications of bioorthogonal lipid chemistry

Although bioorthogonal lipid chemistry was originally developed as a toolkit for interrogating lipid metabolism and lipid–protein interactions, its scope has progressively expanded toward therapeutic applications. Rather than serving exclusively as imaging probes or chemical reporters, bioorthogonal lipids can now function as structural components of drug-delivery systems, programmable prodrugs, and targeting ligands that enhance the selectivity of therapeutic agents.^11,102–104^ Two complementary concepts underpin these emerging applications. First, covalent conjugation of lipids to therapeutic molecules modulates their physicochemical and pharmacokinetic properties, promoting membrane association, self-assembly into nanostructures, and stimulus-responsive drug release. Second, metabolic glycoengineering and related bioorthogonal labelling strategies introduce artificial chemical handles onto cell surfaces, enabling selective recognition by complementary carriers independently of endogenous receptor expression. Together, these advances are redefining the role of lipids from passive delivery components to programmable elements for the engineering of increasingly precise therapeutic systems.

### 5.1 Bioinspired drug delivery platforms

Among lipid-based therapeutic technologies, bioinspired drug delivery systems (DDSs) occupy an intermediate position between conventional nanocarriers, such as liposomes and polymeric micelles, and living cell therapies.^102^ By reproducing selected structural and functional features of biological membranes without incorporating living components, these systems combine the versatility of biomimetic materials with the safety, reproducibility, and manufacturing advantages of fully synthetic platforms. Their modular architecture also enables precise control over composition, size, surface chemistry, and cargo loading, thereby facilitating reproducible pharmacological performance while avoiding many of the regulatory and biosafety challenges associated with cell-based therapies.^102^

In this context, the covalent attachment of lipids to therapeutic molecules has emerged as a widely investigated approach for the development of lipid-based drug delivery systems with enhanced physicochemical characteristics, self-assembly properties, and improved pharmacological behaviour (Fig. 15).^2^ Lipid–drug conjugates (LDCs) take advantage of the inherent amphiphilic nature of lipid moieties to promote the efficient incorporation of therapeutic agents into nanoscale delivery platforms, while also enabling controlled drug release and enhanced bioavailability. Among the most commonly explored strategies, esterification reactions have received significant attention due to the availability of reactive carboxylic acid functionalities in lipids, particularly fatty acids. Through the generation of ester bonds, a broad range of therapeutic compounds, including antibiotics, have been covalently coupled to fatty acids^105^ and squalene-based derivatives.^106^ For example, the conjugation of bromo-functionalized doxorubicin with squalenoic acid resulted in an LDC capable of spontaneously assembling into elongated nanostructures, whereas similar squalene-derived nanosystems have been obtained using paclitaxel as the therapeutic cargo.^105,106^ In addition to ester-based strategies, amide bond formation has been extensively explored as a versatile methodology for the synthesis of LDCs. By employing carbodiimide-mediated coupling chemistry, Paliwal *et al.* successfully linked low-molecular-weight heparin to different fatty acids through the reaction between free amino groups and lipid-derived carboxylic acid groups.^107^ These lipidated derivatives demonstrated improved bioavailability, and their incorporation into PC-based vesicular formulations further enhanced drug protection against biodegradation and promoted improved absorption *in vivo*. Furthermore, stimuli-responsive chemical linkers, including hydrazone bonds, have attracted increasing interest due to their pH-responsive behaviour (Fig. 15B).^108^ Their stability under physiological conditions, combined with their ability to undergo cleavage in mildly acidic environments, supports the design of LDC-based delivery systems that remain stable during circulation and storage while enabling efficient therapeutic release after endosomal uptake.^108^

**Fig. 15:**
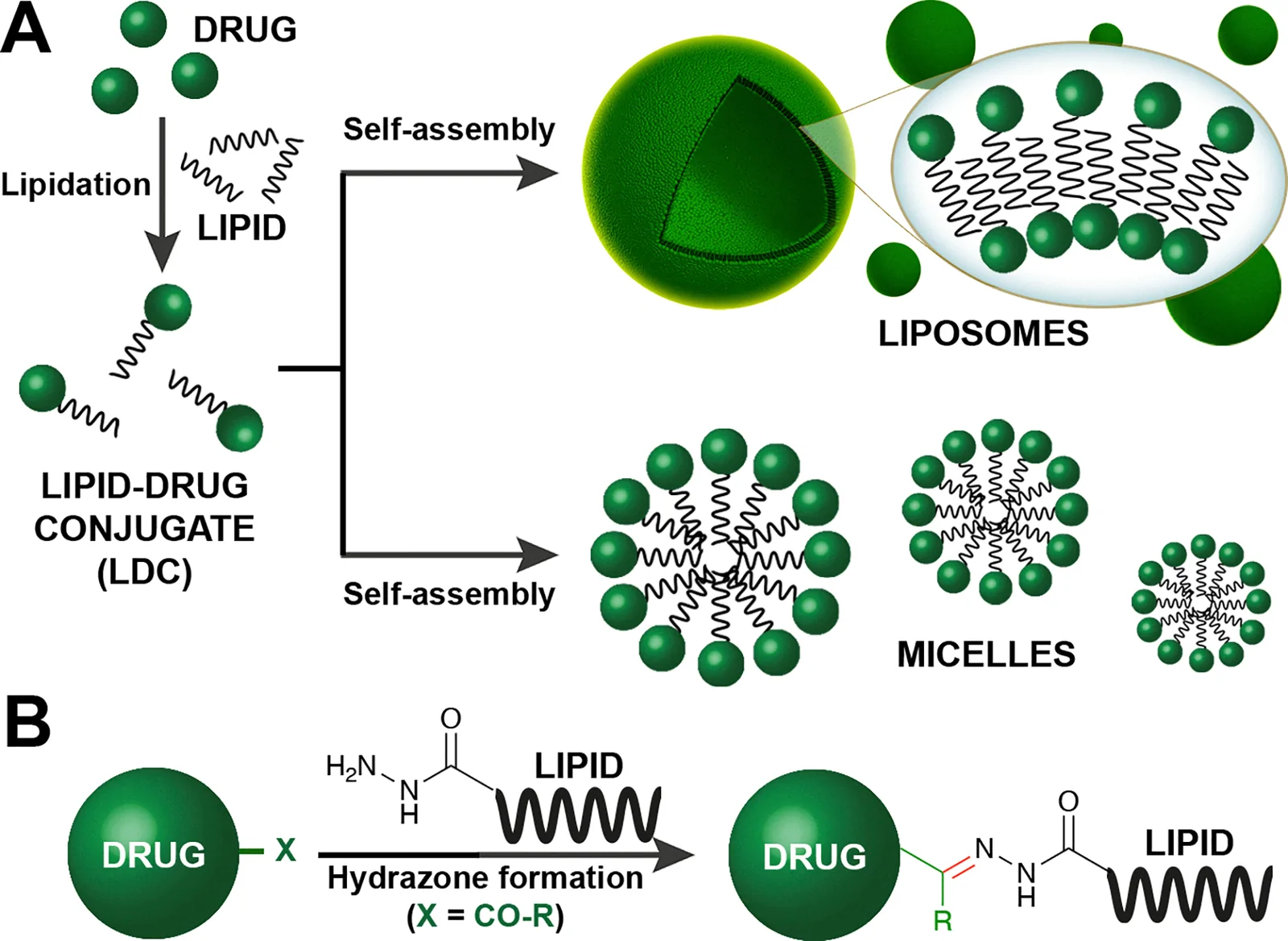
Lipid modification strategies for the development of advanced drug delivery systems. (A) Schematic representation of the generation of drug delivery platforms through the lipidation of the corresponding therapeutic molecule.^2^ This approach involves the covalent attachment of a lipid moiety to the drug, yielding a lipid–drug conjugate (LDC) with amphiphilic properties. These conjugates can spontaneously organize into diverse nanostructures, including liposomes and micelles. (B) Hydrazone-based strategy for the synthesis of LDCs.^108^ Figure adapted from ref. 2 with permission from the Royal Society of Chemistry, copyright 2020.

Bioorthogonal chemistry has emerged as a key enabling technology for these platforms because it provides rapid, highly selective reactions that proceed under physiological conditions without perturbing endogenous biochemical processes. In the context of drug delivery, these reactions enable the site-specific functionalization of nanoparticles, liposomes, extracellular vesicles, and cell membranes with targeting ligands, imaging agents, or therapeutic cargoes while preserving the biological integrity of the carrier.^11^ More importantly, bioorthogonal ligation offers an attractive alternative to conventional receptor-mediated targeting, the effectiveness of which is frequently limited by heterogeneous receptor expression, receptor downregulation, or poor tissue penetration. Instead, metabolically installed chemical reporters create synthetic recognition motifs that can be selectively addressed by complementary bioorthogonal ligands, establishing programmable targeting strategies that are largely independent of endogenous receptor biology.^11^

Unfortunately, several important challenges continue to limit clinical translation. The pronounced heterogeneity of the tumour microenvironment (TME) complicates homogeneous targeting, while immune responses to formulation components such as polyethylene glycol (PEG) and the accelerated blood-clearance phenomenon have stimulated the development of alternative carrier designs, including PEG-free based nanoassemblies.^11^ In addition, the lack of standardized methods for quantifying bioorthogonal membrane functionalization hinders direct comparison between different targeting strategies.

Continued advances in bioorthogonal ligation chemistry, programmable linker design, and biomimetic membrane engineering are expected to improve the selectivity, robustness, and translational potential of lipid-based therapeutic platforms, further expanding the role of bioorthogonal chemistry beyond molecular imaging toward precision nanomedicine.

### 5.2 Lipid-mediated delivery of anticancer agents

The therapeutic efficacy of many anticancer agents is often limited by poor tumour selectivity and dose-limiting toxicity.^11,103,104^ Bioorthogonal chemistry offers an alternative targeting strategy that is largely independent of endogenous receptor biology. Rather than relying on tumour-associated receptors, cancer cells are first metabolically engineered to display non-native chemical functionalities on their surface, which are subsequently recognized by complementary bioorthogonal ligands carried by therapeutic nanoplatforms.^11^ This two-step approach provides a versatile and programmable strategy for enhancing tumour-selective drug delivery.

Among the available metabolic reporters, tetraacetylated *N*-azidoacetylmannosamine (Ac_4_ManNAz) is the most widely employed.^11^ Following cellular uptake, Ac_4_ManNAz enters the sialic acid biosynthetic pathway, resulting in the incorporation of azide-functionalised sialic acids into cell-surface glycoconjugates.^11^ The resulting artificial azide groups can reach densities of approximately 10^7^ per cell, exceeding the abundance of most endogenous membrane receptors by several orders of magnitude and thereby providing highly efficient anchoring sites for complementary bioorthogonal ligands.^11,103^

A representative example was reported by Qiao *et al.*, who developed a DBCO-functionalised low-molecular-weight heparin–quercetin conjugate that spontaneously self-assembled into multifunctional nanocomposites encapsulating both doxorubicin and the photosensitiser zinc phthalocyanine.^103^ Following metabolic priming with Ac_4_ManNAz, the nanoparticles selectively accumulated at the tumour surface through SPAAC-mediated ligation with metabolically installed azide groups. This stable covalent anchoring enabled synergistic chemo-photothermal therapy, illustrating how lipid-mediated self-assembly and bioorthogonal targeting can be integrated within a single therapeutic platform.

Bioorthogonal targeting has also been incorporated into theranostic lipid nanomedicines. Lipid-based micellar systems carrying phthalocyanine photosensitisers can be selectively anchored to metabolically labelled tumour cells through azide–alkyne bioorthogonal ligation prior to optical activation (Fig. 16).^11,104^ This strategy enhances local nanoparticle retention and concentrates photothermal and photoacoustic effects within the TME, thereby improving nanoparticle penetration, intracellular drug delivery, and overall therapeutic efficacy.

**Fig. 16:**
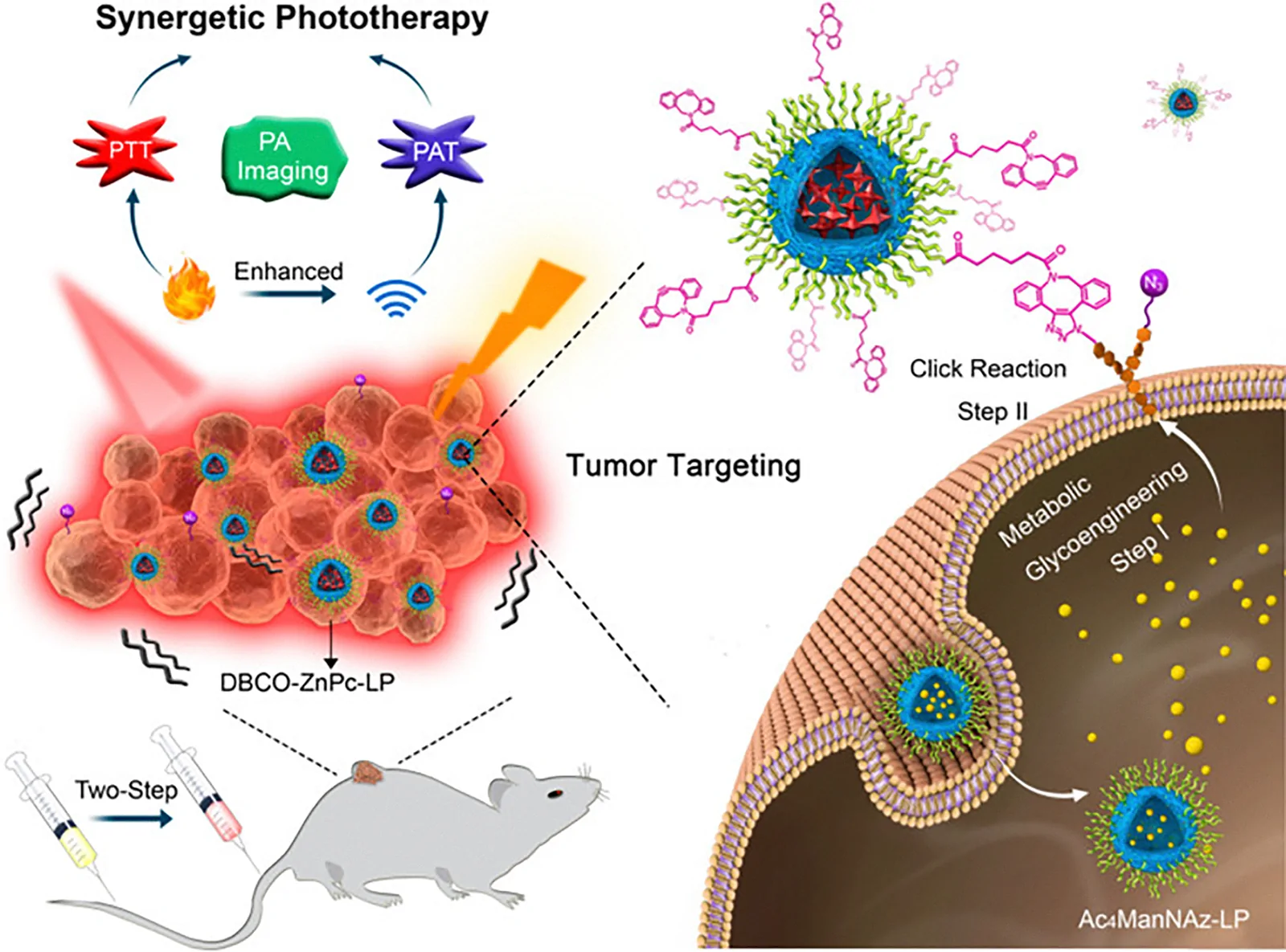
Schematic representation of a tumour-targeting agent activated by bioorthogonal metabolic glycoengineering.^104^ Reproduced from ref. 104 with permission from American Chemical Society, copyright 2017.^104^

The utility of bioorthogonal lipid chemistry extends beyond targeted drug delivery to monitoring therapeutic responses. Luo *et al.* employed the clickable choline analogue ProCho, which is metabolically incorporated into membrane-forming PCs and subsequently visualised through fluorescent azide click labelling.^109^ Reductions in phospholipid biosynthesis became detectable within hours of cisplatin treatment, preceding conventional measurements of cell viability or apoptosis. Moreover, ProCho labelling proved more sensitive than an analogous fluorescent glucose uptake probe, demonstrating that phospholipid metabolism provides an early pharmacodynamic readout of treatment response. Because labelling can be quantified at single-cell resolution in both monolayer cultures and tumour spheroids, this strategy also offers opportunities for identifying drug-resistant subpopulations and evaluating patient-derived samples *ex vivo*.

Despite these encouraging advances, several challenges continue to limit the clinical translation of bioorthogonal lipid-based targeting strategies. Achieving homogeneous metabolic labelling throughout solid tumours remains difficult because the dense and heterogeneous TME restricts the penetration of both nanocarriers and metabolic precursors, resulting in incomplete installation of bioorthogonal handles.^11,66^ Systemic administration of metabolic reporters further introduces pharmacokinetic challenges, including rapid clearance, limited solubility, and nonspecific incorporation into healthy tissues. To address these limitations, nanoparticle-mediated precursor delivery, stimuli-responsive metabolic reporters, extracellular vesicles, and implantable reservoirs are being explored to improve tumour-selective labelling.^11^

Additional challenges arise from the kinetics of metabolic labelling, which typically requires hours to days before sufficient numbers of bioorthogonal groups accumulate on the cell surface, thereby limiting applications requiring rapid intervention. From the perspective of ligation chemistry, copper-free reactions such as SPAAC remain the preferred choice for live-cell and *in vivo* applications, whereas CuAAC continues to be valuable for analytical and fixed-cell studies.^109^

Finally, an important conceptual limitation concerns the choice of metabolic precursor. To date, metabolic glycoengineering has proven substantially more effective for tumour targeting than analogous strategies based on lipid metabolism, largely because cell-surface glycans exhibit greater tumour-specific remodelling than membrane phospholipids.^11^ The development of lipid precursors that undergo preferential metabolic incorporation in defined tumour types therefore represents an important future objective, with the potential to extend bioorthogonal targeting beyond glycans and enable direct exploitation of tumour-specific lipid metabolism for precision drug delivery.

## 6. Conclusions and future outlook

This review has highlighted the remarkable evolution of lipid bioorthogonal chemistry from a collection of proof-of-concept transformations into a mature and increasingly integrated discipline encompassing lipid synthesis, visualization, mechanistic interrogation, and therapeutic exploitation. Despite the diversity of applications described, these advances are unified by a common design principle: the replacement of perturbing reporters with minimal, chemically inert handles that can subsequently be converted into detectable or functional entities through rapid and selective reactions performed in biological environments.^2,5^ Whether based on azides, terminal alkynes, tetrazine–dienophile pairs, small vibrational tags, or photoactivatable groups such as diazirines, this strategy has fundamentally reshaped the experimental investigation of lipid biology. Rather than representing isolated chemical innovations, these approaches collectively provide a framework for manipulating and observing lipid behaviour while preserving native biological context.

Building upon this foundation, the capabilities of the field have expanded dramatically. Membrane-forming lipids can now be generated through abiotic synthesis and induced to self-assemble within living systems, providing unprecedented opportunities to investigate and engineer membrane biogenesis.^8^ Imaging methodologies have progressed beyond simply localization to enable analysis of lipid chemical identity, membrane leaflet distribution, intracellular trafficking, and dynamic behaviour, while label-free vibrational approaches have extended lipid imaging to living organisms.^9,49,68,70,72–74^ In parallel, photoaffinity labelling combined with chemoproteomic workflows has enabled systematic mapping of lipid–protein interaction landscapes at the proteome scale, uncovering molecular networks that were previously inaccessible.^99^ More recently, these chemical strategies have begun to influence therapeutic development, supporting targeted drug delivery, stimulus-responsive lipid systems, and approaches for manipulating lipid metabolism in disease contexts.^103^

The remaining challenges are as informative as the achievements and reveal several recurring limitations across the field. The most pervasive is insufficient molecular and spatial selectivity. Metabolic precursors are often distributed across multiple downstream lipid pathways, making it difficult to distinguish abundant structural lipids from discrete signalling pools with specialised functions.^9,42^ Improving metabolic precision through enzyme-specific substrates, organelle-targeted probes, or engineered biosynthetic pathways therefore remains a central objective. A second challenge is the persistent trade-off between minimal perturbation and analytical performance. The fastest bioorthogonal reactions frequently rely on larger reactive partners, photo-crosslinking captures only a limited fraction of transient interactions, and even comparatively small fluorophores can influence membrane organization, trafficking, or protein function.^37,101^ Continued development of smaller, faster, and more biologically compatible chemistries will therefore be essential.

Spatial and temporal resolution also remains a significant bottleneck. Although substantial progress has been achieved through super-resolution microscopy, expansion microscopy, and vibrational imaging, resolving nanoscale lipid organization, distinguishing the two leaflets of individual membrane bilayers, and following rapid lipid dynamics in living cells remain technically demanding.^54^ Furthermore, while many bioorthogonal lipid approaches have become routine in cultured cells, their application in intact tissues and whole organisms remains comparatively underdeveloped. In contrast to glycobiology, where metabolic labelling has already become a widely established *in vivo* methodology, lipid bioorthogonal chemistry has yet to achieve similar levels of robustness, selectivity, and general applicability.^6,75^

Looking ahead, the next major advances in the field are unlikely to arise from the discovery of a single transformative reaction. Instead, progress will likely emerge from the integration of complementary technologies into unified experimental platforms. The combination of programmable lipid synthesis, multiplexed imaging, spatially resolved lipidomics, chemoproteomic interaction mapping, and targeted therapeutic delivery could ultimately enable workflows in which lipids are synthesised, visualised, perturbed, and functionally characterised within the same biological system. Coupling these approaches with advances in artificial intelligence for image and omics analysis, high-throughput screening, and increasingly biocompatible reaction platforms will further accelerate the transition from descriptive lipid mapping toward predictive and programmable lipid biology. If the past two decades established bioorthogonal chemistry as an indispensable tool for investigating lipids in living systems, the coming decade may transform these approaches into integrated platforms for controlling lipid function, revealing new mechanisms of disease, and enabling precision therapeutic strategies.

## Author contributions

Brenda Portasany: writing – original draft, review & editing. Federica A. Souto-Trinei: writing – original draft, review & editing. Roberto J. Brea: writing – original draft, review & editing, supervision.

## Conflicts of interest

There are no conflicts to declare.

## Data Availability

No primary research results, software or code have been included and no new data were generated or analysed as part of this review.
